# Thermal acclimation fails to confer a carbon budget advantage to invasive species over natives

**DOI:** 10.1093/plphys/kiaf574

**Published:** 2025-11-10

**Authors:** Thibaut Juillard, Christoph Bachofen, Maxwell Bergström, Marco Conedera, Mattéo Dumont, Jean-Marc Limousin, Arianna Milano, Gianni Boris Pezzatti, Alberto ViIagrosa, Charlotte Grossiord

**Affiliations:** Plant Ecology Research Laboratory PERL, School of Architecture, Civil and Environmental Engineering ENAC, EPFL, Lausanne CH-1015, Switzerland; Community Ecology Unit, Swiss Federal Institute for Forest, Snow and Landscape Research WSL, Birmensdorf CH-8903, Switzerland; Plant Ecology Research Laboratory PERL, School of Architecture, Civil and Environmental Engineering ENAC, EPFL, Lausanne CH-1015, Switzerland; Community Ecology Unit, Swiss Federal Institute for Forest, Snow and Landscape Research WSL, Birmensdorf CH-8903, Switzerland; Plant Ecology Research Laboratory PERL, School of Architecture, Civil and Environmental Engineering ENAC, EPFL, Lausanne CH-1015, Switzerland; Community Ecology Unit, Swiss Federal Institute for Forest, Snow and Landscape Research WSL, Birmensdorf CH-8903, Switzerland; Community Ecology Unit, Swiss Federal Institute for Forest, Snow and Landscape Research WSL, Birmensdorf CH-8903, Switzerland; Génie énergétique et génie de l'environnement GEn, Institut National des Sciences Appliquées de Lyon, INSA, Villeurbanne 69100, France; Centre d'Ecologie Fonctionnelle et Evolutive CEFE, University Montpellier, CNRS, EPHE, IRD, Montpellier 34090, France; Plant Ecology Research Laboratory PERL, School of Architecture, Civil and Environmental Engineering ENAC, EPFL, Lausanne CH-1015, Switzerland; Community Ecology Unit, Swiss Federal Institute for Forest, Snow and Landscape Research WSL, Birmensdorf CH-8903, Switzerland; Community Ecology Unit, Swiss Federal Institute for Forest, Snow and Landscape Research WSL, Birmensdorf CH-8903, Switzerland; Mediterranean Center for Environmental Studies (CEAM Foundation). Joint Research Unit University of Alicante-CEAM, University of Alicante, Sant Vicent del Raspeig, Alicante 03690, Spain; CEAM-Department de Ecologia, Universitat d'Alacant, POB 99, Alacant E-03080, Spain; Plant Ecology Research Laboratory PERL, School of Architecture, Civil and Environmental Engineering ENAC, EPFL, Lausanne CH-1015, Switzerland; Community Ecology Unit, Swiss Federal Institute for Forest, Snow and Landscape Research WSL, Birmensdorf CH-8903, Switzerland

## Abstract

Both native and invasive plants can adjust photosynthesis and respiration when exposed to warmer temperatures. However, it is uncertain whether invasive plants are more plastic and exhibit higher acclimation to rising temperatures than native ones, a trait that could contribute to their invasive behavior in novel environments. We compared the capacity of the windmill palm (*Trachycarpus fortunei*), a highly invasive species in central Europe, and 2 native co-occurring species, European holly (*Ilex aquifolium*) and small-leaved linden (*Tilia cordata*), to acclimate photosynthesis and respiration to air temperature changes using a 2-yr-long transplant experiment across Europe (mean temperatures ranging from 8.4 to 21.8 °C). We measured the optimal temperature of photosynthesis (T_opt_), the assimilation at optimal temperature (A_opt_), the thermal breadth of photosynthesis (T_80_), the respiration at 25 °C (R_25_), the temperature sensitivity of respiration (Q_10_), and simulated the whole-plant carbon budget. For all species, T_opt_, A_opt,_ and T_80_ increased with warming, while R_25_ decreased in the native species and Q_10_ decreased in the invasive species only. Consequently, acclimation enhanced the carbon budget of the invasive and native plants in the warm and hot sites. The invasive palm had a similar or lower acclimation capacity than other species and a lower but constant carbon budget across the European temperature gradient. Our work reveals that not all invasive plants exhibit greater photosynthetic plasticity than native ones, suggesting that temperature-driven enhancement of their carbon budget may play a limited role in future invasion processes.

## Teaser

The invasive windmill palm shows no greater ability than native trees to adjust photosynthesis to warming, suggesting temperature acclimation alone does not drive plant invasions.

## Introduction

Global warming promotes the spread of invasive plant species worldwide, representing one of the most important threats to plant biodiversity (Walther et al. 2009; Liu et al. 2017; Pyšek et al. 2020). Among possible factors, long-lived invasive plants can outperform native species under a warmer climate as they tend to be adapted to broader air temperature (T_air_) ranges (e.g. Higgins and Richardson 2014; Finch et al. 2021; Turbelin and Catford 2021), can extend their growing seasons more significantly (e.g. Fridley 2012; Juillard et al. 2024), and acclimate various functional traits more extensively (e.g. Davidson et al. 2011; Gioria et al. 2023). Higher carbon (C) uptake and plant productivity following a greater extent of thermal acclimation of photosynthesis and respiration may, in turn, enhance species competitiveness (Duan et al. 2013; Yu et al. 2019; Grossiord et al. 2022). Still, few studies investigated photosynthetic and respiratory thermal acclimation in the context of species invasiveness (Verlinden and Nijs 2010; Godoy et al. 2011; Ripley et al. 2020), with none addressing if invasive plants are more plastic than native ones with increasing temperature. Yet, understanding and predicting these processes would help reduce uncertainties in climate-vegetation models, where acclimation is often simplified or fully neglected (Crous et al. 2022) and would allow finding appropriate conservation strategies for forests highly susceptible to invasion as a consequence of global warming.

Plants generally acclimate rapidly to higher T_air_ (within 1 mo; e.g. Kattge and Knorr 2007; Kumarathunge et al. 2019) by increasing their optimal temperature for photosynthesis (T_opt_, i.e. the temperature at which the net assimilation (A_net_) reaches its maximum), its corresponding optimal net assimilation value (A_opt_), and their thermal breadth (T_80_), which represents the temperature range where photosynthesis reaches >80% of its maximum (Yamori et al. 2014), thereby maintaining C gain despite warmer air (Kruse et al. 2019; Vico et al. 2019; Choury et al. 2022). Thermal acclimation of photosynthesis also depends on stomatal conductance (g_s_) (Kruse et al. 2019; Kullberg et al. 2023) and phenology (Grossiord et al. 2022), which vary interannually (Petrik et al. 2022; Didion-Gency et al. 2024), leading to substantial uncertainties in plant's thermal acclimation capacity. Shifts in A_opt_, T_opt_, and T_80_ are mainly driven by an increase in the maximum catalytic activity of Rubisco (V_C,max_) and the maximum ratio of electron transport (J_max_) (Medlyn et al. 2002). Further, V_C,max_ and J_max_ are both limited by nitrogen availability, therefore, thermal acclimation of photosynthesis also depends on nitrogen allocation, for which invasive plants tend to be more plastic than native ones (Feng and Fu 2008; Funk et al. 2013). Accordingly, invasive species may exhibit greater plasticity in the V_C,max_-to-J_max_ ratio, enabling a closer adjustment of photosynthetic capacity to rising air temperature than natives.

In parallel, acclimation to higher T_air_ can also involve lowering the rate of respiration at 25 °C (R_25_) and the respiration yield every 10 °C (Q_10_) (Atkin and Tjoelker 2003; Atkin et al. 2005), thereby limiting C loss at high temperatures as respiration increases exponentially (Atkin and Tjoelker 2003; Way and Yamori 2014; Crous et al. 2022). Plants with higher net assimilation acclimation can show higher respiration acclimation (Dusenge et al. 2019), although respiration can also acclimate more extensively to T_air_ than net assimilation (Campbell et al. 2007; Way and Oren 2010; Crous et al. 2022). Just as for photosynthesis, the duration of exposure to changed temperatures can influence respiration acclimation. Typically, acclimation of R_25_ to a particular T_air_ occurs during tissue development, while Q_10_ varies more rapidly with seasonal changes in ambient T_air_ (Atkin et al. 2005). Some studies found that respiration tends to acclimate universally among plant species (Crous et al. 2022), and others reported acclimation to be species-specific independently of biomes or functional groups (e.g. evergreen *vs*. deciduous) (Slot and Kitajima 2015).

Respiratory rates are closely linked to leaf nitrogen content and specific leaf area (Lee et al. 2005; Xu et al. 2007), traits often higher in invasive species than in native ones (Leishman et al. 2007). Because higher respiration at a given temperature can lead to greater C loss under warming, the capacity to acclimate respiration could be particularly beneficial for species with high nitrogen and metabolic demand. However, temperature-induced reductions in respiratory rates have rarely been investigated in invasive species, leaving the extent and ecological significance of such acclimation largely unknown. Therefore, invasive species may exhibit a greater extent of respiratory thermal acclimation than co-occurring native species, potentially allowing them to maintain a more favorable C balance under rising temperatures.

In this study, we compared the acclimation of leaf photosynthesis and respiration of the invasive windmill palm (*Trachycarpus fortunei*), a palm native to South-eastern China, with 2 co-occurring natives growing in the southern Alps in a sub-mediterranean climate i.e. the evergreen European holly (*Ilex aquifolium*) and deciduous small-leaved linden (*Tilia cordata*). Since the 2000s, *T. fortunei* has been colonizing natural forests worldwide, impacting the regeneration of native species (Fehr et al. 2023 ). In the southern Alps, the spread of *T. fortunei* and other non-native thermophilic evergreen species has been facilitated by a high propagule pressure from cultivated individuals in settlements, and is further correlated with the rise in T_air_ since the 1970s (ΔMAT: +1.7 °C) (Walther et al. 2007; Conedera et al. 2018; Fehr et al. 2023). As such, *T. fortunei* is suspected to benefit from warmer temperatures to outcompete its native competitors, but whether this includes enhanced photosynthetic and respiratory thermal acclimation is unknown. Supporting this, Kruse et al. (2019) observed extended acclimation of T_opt_, A_opt_, and g_s_ with T_air_ in date palms, resulting in a close alignment of photosynthesis with prevailing temperatures.

Using a transplant experiment in 5 sites covering a large range of mean T_air_ across Europe, we measured the responses of photosynthesis and respiration to temperature over 2 yr. A soil-plant-atmosphere continuum (SPAC) model was used to estimate the impact of temperature acclimation on the leaf and whole-plant C budget over an entire growing season. We hypothesized that (ⅰ) the invasive *T*. *fortunei* acclimates more extensively than the native species to increased T_air_, leading to higher A_opt_, T_opt_, and T_80_. Similarly, (ⅱ) the invasive *T*. *fortunei* displays lower R_25_ and Q_10_ than native species with higher T_air_ and that, as such, (ⅲ) *T. fortunei* assimilates more C than native species in warmer sites.

## Results

### Photosynthetic and respiration responses to air temperature

**Figure 1.**
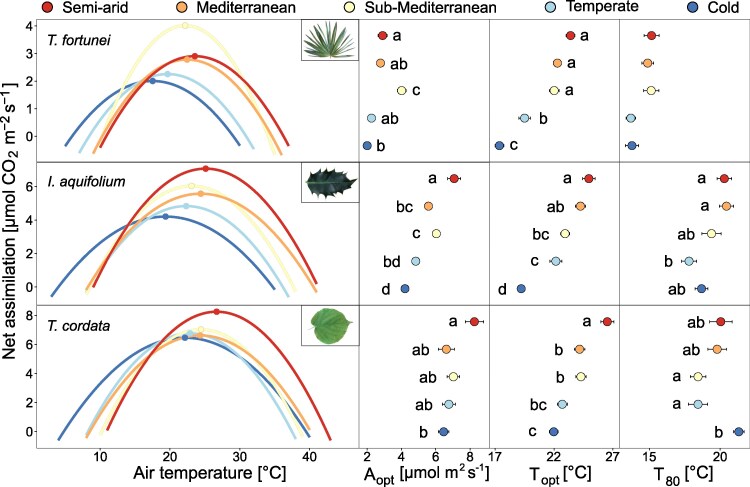
Assimilation-temperature response curves for the summer months (mean of May–October) of both years (2022–2023) for *T. fortunei* (*R*^2^ = 0.70), *I. aquifolium* (*R*^2^ = 0.66), and *T. cordata* (*R*^2^ = 0.83) at the 5 experimental sites (indicated with colors from blue to red going from the coldest to the warmest). On the right panels, the mean and standard errors of A_opt_, T_opt_, and T_80_ at each site are shown (*n* = 4–10 individuals per species). Different letters indicate significative differences (*P* < 0.05) between sites for each species based on Tukey's HSD test.

In both years, A_opt_, T_opt_, and T_80_ differed between species and climates (*P* < 0.001 for A_opt_ and T_opt_ and *P* < 0.05 for T_80_; Fig. 1 and Supplementary Table S1), with generally higher values in the warmest site and lower values in the coldest one (Supplementary Fig. S1 and Table S2). In all sites, A_opt_ and T_opt_ were lower in *T. fortunei* (2.86 *µ*mol m^2^ s^−1^ and 21.3 °C on average, respectively) than in *I. aquifolium* (5.5 *µ*mol m^2^ s^−1^ and 22.7 °C on average, respectively) and *T. cordata* (6.96 *µ*mol m^2^ s ^1^ and 23.9 °C on average, respectively), even in the reference sub-Mediterranean climate where *T. fortunei* is highly invasive (*P* < 0.001; Fig. 1). Similarly, *T. fortunei* had a lower T_80_ (11.1 °C on average) than *I. aquifolium* (13.9 °C on average) and *T. cordata* (15.2 °C on average) in all sites (Fig. 1).

**Figure 2.**
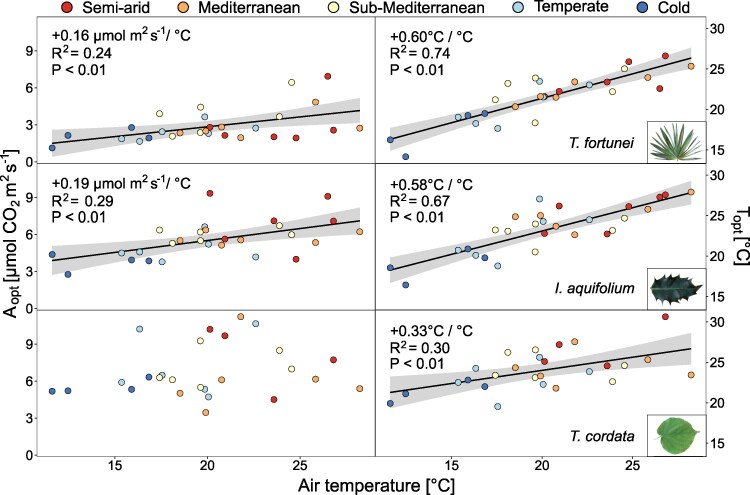
A_opt_ and T_opt_ (*n* = 4–10 individuals per species) averaged by campaigns in relation to air temperature of the 2 wk preceding the measurements for *T*. *fortunei*, *I*. *aquifolium*, and *T*. *cordata*. Colors represent climates from blue to red, from the coldest to the warmest. Linear regression lines (ordinary least squares) were added when significant.

**Figure 3.**
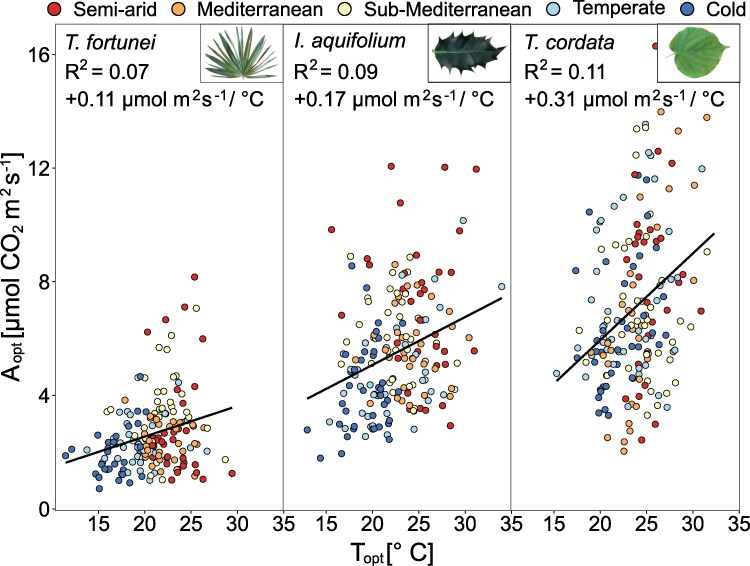
Assimilation at the optimal temperature (A_opt_) in function of the optimal temperature of photosynthesis (T_opt_) (*n* = 4–10 individuals per species) in *T. fortunei*, *I. aquifolium*, and *T. cordata* during all campaigns in 2022 and 2023. Colors represent sites from blue to red, from the coldest to the warmest. Linear regression lines (ordinary least squares) were added when significant (*P* < 0.01 for all species).

T_opt_ was strongly correlated with the mean T_air_ of the 2 preceding weeks, whereas the relationship between A_opt_ and T_air_ was weaker (Fig. 2), suggesting that photosynthetic acclimation in T_opt_ was more pronounced than in A_opt_. The relationship between mean T_air_ and T_opt_ was significant for all species (*P* < 0.01), but steeper for the 2 evergreen species than for the deciduous one (+0.60, +0.55, and +0.33 °C T_opt_ per °C mean T_air_ for *T*. *fortunei*, *I*. *aquifolium*, and *T*. *cordata*, respectively). The same relationship with A_opt_ was significant in *T. fortunei* (+0.16 *µ*mol m^2^ s^−1^ per °C; *P* < 0.001) and *I. aquifolium* (+0.19 *µ*mol m^2^ s^−1^ per °C; *P* < 0.001), but not for *T. cordata* (Fig. 2). Air temperature and T_80_ were only correlated in *T. fortunei* (+0.19 °C per °C air temperature, *R*^2^ = 0.25, *P* < 0.01; Supplementary Fig. S2). A_opt_, T_opt_, and T_80_ were weakly positively correlated in all species (*P* < 0.05; Fig. 3 and Supplementary Fig. S2), except for T_opt_ and T_80_ in *T. fortunei*. In *T. fortunei*, A_opt_ increased by 0.12 *µ*mol m^2^ s^−1^ per °C positive shift in T_opt_. The same correlation was steeper in *I. aquifolium* and *T. cordata* (+0.23 and +0.27 *µ*mol m^2^ s^−1^ per °C shift in T_opt_, respectively; Fig. 3).

**Figure 4.**
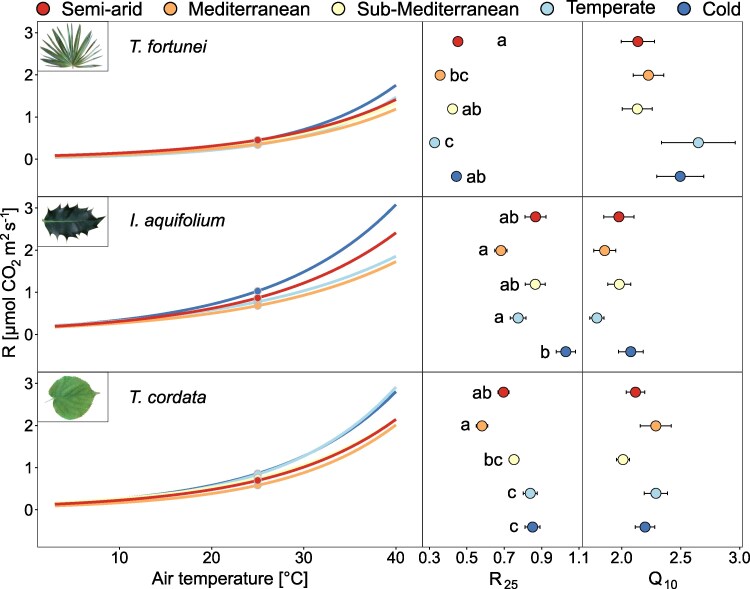
Respiration-temperature response curves for the summer months (mean of May–October) of both years (2022–2023) for *T. fortunei* (*R*^2^ = 0.44), *I. aquifolium* (*R*^2^ = 0.51), and *T. cordata* (*R*^2^ = 0.53) at the 5 experimental sites (indicated with colors from blue to red, from the coldest to the warmest). The right panels show the mean and standard errors of R_25_ and Q_10_ at each site (*n* = 4–10 individuals per species). Different letters indicate significant differences (*P* < 0.05) between the sites based on Tukey's HSD test.

**Figure 5.**
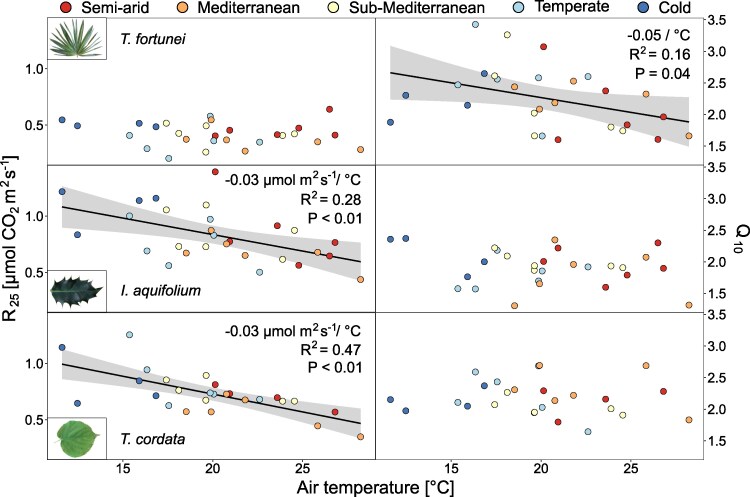
R_25_ and Q_10_ (*n* = 4–10 per species) averaged by campaigns in relation to the air temperature of the 2 wk preceding the measurements for *T. fortunei*, *I. aquifolium*, and *T. cordata*. Colors represent sites from blue to red, from the coldest to the warmest. Linear regression lines (ordinary least squares) were added when significant.

R_25_ varied across sites and species (*P* < 0.001; Fig. 4 and Supplementary Table S1) with higher values in the colder sites, except in *T*. *fortunei*. On average, the R_25_ of *T*. *fortunei* (0.40 *µ*mol m^2^ s^−1^) was half of the R_25_ in *I. aquifolium* and *T. cordata* (0.85 and 0.75 *µ*mol m^2^ s^−1^, respectively) (Fig. 4). R_25_ decreased with increasing mean T_air_ of the 2 preceding weeks in *I. aquifolium* and *T. cordata* (−0.03 *µ*mol m^2^ s^−1^/°C in both species, *P* < 0.01; Fig. 5) but not in *T. fortunei,* suggesting limited temperature acclimation in that species. On the other hand, while Q_10_ was different between species and sites (*P* < 0.001; Fig. 4 and Supplementary Table S1), Q_10_ decreased with increasing mean T_air_ only in *T. fortunei* (Fig. 5).

### Modeled carbon uptake

The SPAC model produced more precise predictions for *I. aquifolium* and *T. cordata* than for *T. fortunei* (*R*^2^ = 0.61, 0.66, and 0.17; NSE = 0.47, 0.62, and 0.11, respectively). Hence, C_leaf_ comparison between sites was impacted by the low precision of the model for *T*. *fortunei* (*R*^2^ = 0.17, Supplementary Table S3) which decreased the statistical power of our results. Still, bias was low in all species (−0.61, 1.41, and 1.72%, respectively, Supplementary Table S3), indicating a high accuracy of the model.

**Figure 6.**
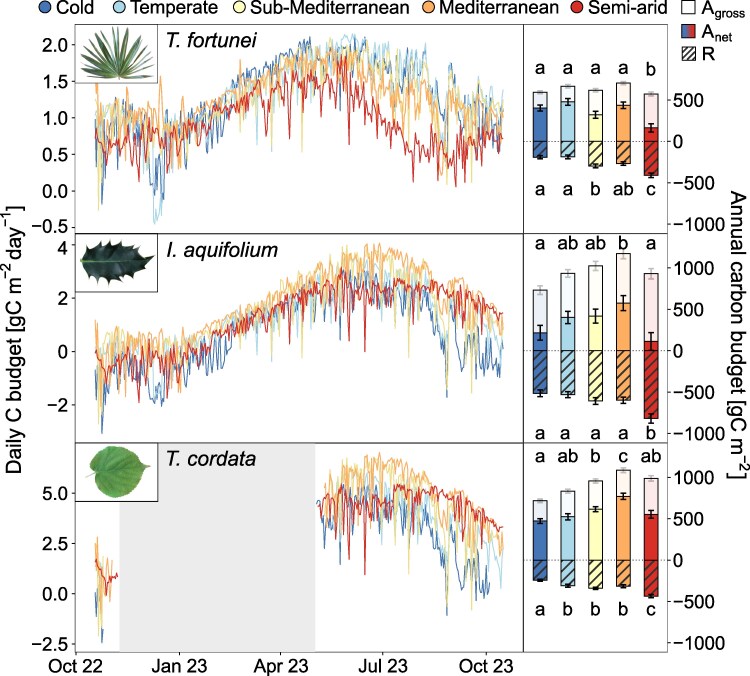
Daily mean leaf-level C budget in *T. fortunei*, *I. aquifolium*, and *T. cordata* at the 5 experimental sites from 15th October 2022 to 15th October 2023. The right panels show the annual leaf-level C uptake for each site over the same period. Gross assimilation (A_gross_) corresponds to the total length of the bars above 0, net assimilation (A_net_) corresponds to the plain bars, whereas respiration (R) bars are dashed in black. Standard error bars indicate the uncertainty of J_max,25_, V_C,max,25_, R_25_, and Q_10_ (*n* = 37–57). Different letters indicate significant differences (*P* < 0.05) between the sites based on Tukey's HSD test.

Overall, the model revealed substantial differences in the annual leaf-level C uptake (C_leaf_) between species (i.e. 360, 345, and 587 gC m^−2^ on average for *T*. *fortunei*, *I*. *aquifolium*, and *T*. *cordata*, respectively; Fig. 6) and climates (*P* < 0.001), as well as an interaction between species and climate (*P* = 0.016, Supplementary Table S4). At the reference site, *T. fortunei* had the lowest C_leaf_ (321 gC m^−2^) compared with *I. aquifolium* and *T*. *cordata* (418 and 615 gC m^−2^, respectively; Fig. 6). However, *T*. *fortunei* maintained a relatively constant C_leaf_ between the sites except at the warmest site, where a high R resulted in a lower C_leaf_ (Fig. 6 and Supplementary Table S5). In contrast, both native species show the lowest C_leaf_ at the coldest site, a progressively higher C_leaf_ until a peak at the Mediterranean site, and a subsequent decrease at the warmest site (i.e. semi-arid) (Fig. 6). Notably, *T*. *cordata* was the only species that kept a similarly high C_leaf_ at the warmest site as at the reference sites despite higher R (Fig. 6).

Total leaf area did not vary largely between sites, apart for *T*. *fortunei*, which had a larger leaf area in the 3 warmest sites compared with the 2 colder ones (i.e. because of frost damage in winter 2022–2023; Supplementary Fig. S3). As a consequence, total C uptake (C_tot_) showed similar patterns as C_leaf_ across sites for all species (i.e. peaking at the Mediterranean site; Supplementary Fig. S4) with higher C_tot_ in *T. cordata* (i.e. because of its higher leaf area; Supplementary Fig. S5) compared with the other species. In contrast to C_leaf_, *T*. *fortunei* had lower C_tot_ in the 2 coldest sites because of reduced leaf area.

**Figure 7.**
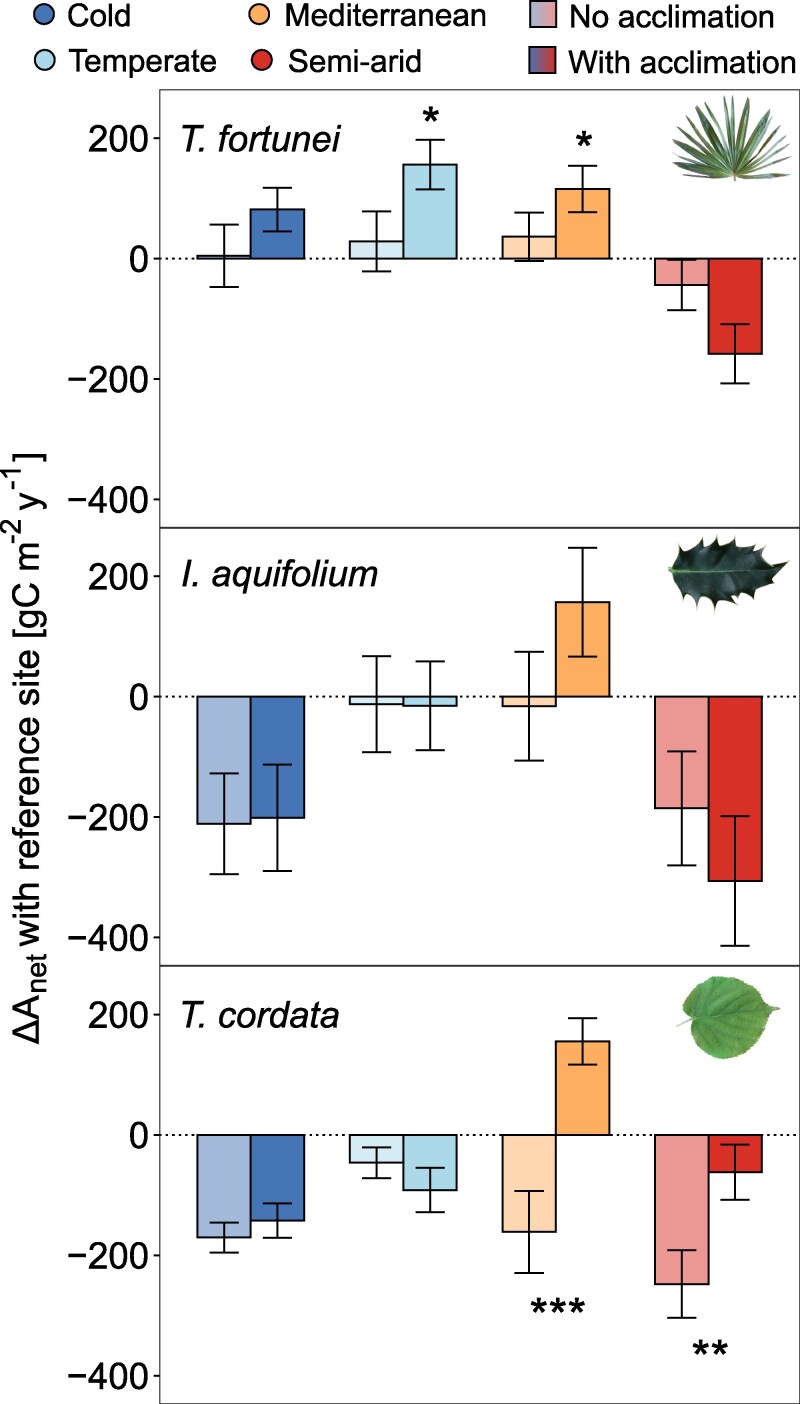
Differences between the modeled annual leaf-level C uptake (ΔA_net_) at every site and the reference site (sub-Mediterranean). Positive values indicate higher annual leaf-level C uptake in the respective sites compared with the reference one. For each site, the lighter bars show results obtained with nonacclimated physiological traits (i.e. the traits from the reference site), while the darker bars correspond to the outputs of the model with acclimated physiology. Standard error bars indicate the uncertainty of modeled J_max,25_ and V_C,max,25_, R_25_, and Q_10_ (*n* = 37 to 57). Significant differences between acclimated and nonacclimated simulations are represented with stars (*P < 0.05; **P < 0.01; ***P < 0.001).

Acclimation of photosynthesis and respiration contributed strongly to the variation in C_leaf_. Without considering acclimation, the C_leaf_ of *T*. *fortunei* would have decreased at the temperate and Mediterranean sites (*P* < 0.05; Fig. 7 and Supplementary Table S6) and remained similar to the C_leaf_ with acclimation at more extreme climates (cold and semi-arid conditions). Similarly, *T*. *cordata* would have had a significantly lower C_leaf_ at the Mediterranean and semi-arid sites (*P* < 0.01; Fig. 7). Contrastingly, acclimation did not modify the C_leaf_ in *I. aquifolium* (Fig. 7).

## Discussion

### Photosynthetic and respiratory acclimation is not higher in invasive palms than in native species

Against our initial hypothesis, the invasive *T*. *fortunei* did not show a higher photosynthetic acclimation than the native species, especially compared with the evergreen *I*. *aquifolium* that showed a similar thermal plasticity (Fig. 2). All species had higher T_opt_ in the warmer sites than the colder ones (Fig. 2), but the 2 evergreen species showed similar T_opt_ acclimation rates at almost double that of the deciduous one (about +0.6 °C vs. +0.3 °C per degree T_air_; Fig. 3). Such differences between functional groups can be explained by the higher need for species with long-lasting tissues to tolerate a broader range of thermal conditions (Yamori et al. 2014). Despite those differences between functional groups, all our shifts in T_opt_ were within the range of +0.3 to 0.8 °C per degree T_air_, as found in most studies on leaf thermal acclimation (e.g. Kumarathunge et al. 2019; Choury et al. 2022; Crous et al. 2022). The T_opt_ shifts were probably driven by changes in the ratio between V_C,max_ and J_max_, as commonly found in other C3 species (Kattge and Knorr 2007; Smith and Dukes 2017), even if more work would be needed to confirm this.

Several studies have highlighted the important role of stomatal aperture in driving shifts of A_opt_ with temperature, especially compared with T_opt_, which is generally less affected by stomatal closure (Kruse et al. 2019; Akaji et al. 2024; Slot et al. 2024). In our study, plants were well-watered, and VPD was maintained as low as technically possible during the measurements, allowing us to assess the thermal acclimation of A_opt_ largely without the bias of increasing stomatal closure, which may explain the stronger relationship between T_air_ and T_opt_ than between T_air_ and A_opt_ (Fig. 2). These results are far-reaching as they suggest that VPD effects on stomata may mask the true acclimation potential of A_opt_, which could be driven by higher enzymatic activity in warmer environments (Berry and Bjorkman 1980) as well as the thermal acclimation of Rubisco activase and chloroplast electron transport under heat stress (Scafaro et al. 2023). Still, similarly to T_opt_, *T*. *fortunei* did not show larger A_opt_ shifts than the other species (Fig. 2). Higher T_opt_ also led to higher A_opt_, and for every °C change in T_opt_, A_opt_ only increased by 0.11 *µ*mol m^2^ s^−1^ in the invasive species, while it increased up to 3 times more for *T. cordata* (Fig. 3). However, *T*. *fortunei* was the only species increasing its T_80_ (i.e. thermal breadth of photosynthesis) with T_air_ (+0.19 °C per °C air temperature; Supplementary Fig. S3), which could advantage the palm under extended diel air temperature variability in warmer climates. These findings are contrary to the existing literature that found that T_80_ decreases with T_air_. Slot and Winter (2017) found an increase in T_80_ accompanied by the rise in the J_max_:V_C,max_ ratio. Hence, a higher T_80_ could be either related to a relative higher J_max_, or lower V_C,max_. However, the authors observed that T_80_ and the J_max_:V_C,max_ were decreasing with warming and not increasing as in *T*. *fortunei*. Some authors attributed a higher V_C,max_ (i.e. a decrease in the J_max_:V_C,max_ ratio) with warming to a counteraction against the increasing photorespiration (Smith and Keenan 2020; Dusenge et al. 2024). One explanation for our results could be that the invasive palm did not regulate photorespiration due a high light tolerance and the shading conditions of our experiment.

Contrary to our initial hypothesis, R_25_ in *T*. *fortunei* did not decrease with higher T_air_, showing no evidence of acclimation (Figs. 4 and 5). Wang et al. (2020) observed that V_C,max_ and R_25_ were decreasing with increasing T_air_ as less active Rubisco is needed for a given value of V_C,max_ at higher temperatures, decreasing the respiratory costs for Rubisco turnover and therefore R_25_. As R_25_ of *T*. *fortunei* remained relatively constant across temperatures, it supports the notion that V_C,max_ remained stable as well in that species, but we did not measure V_C,max_ across temperatures to confirm this. The constant R_25_ may also indicate a lack of acclimation pressure, as at temperatures below 30 °C, respiratory rates were low in this species (Fig. 4). This corroborates that palms can display lower foliar respiration rates than dicot species below 35 °C (Cavaleri et al. 2008). In contrast to *T*. *fortunei*, in the 2 native species, R_25_ decreased with higher T_air_ (−0.03 *μ*mol CO_2_ m^−2^ s^−1^ per degree T_air_ on average; Fig. 5), with similar rates as in other studies (e.g. Reich et al. 2016; Zhu et al. 2021) following the consistent shifts of R_25_ with T_air_ found in a wide range of species and diverse experimental designs (Slot and Kitajima 2015; Crous et al. 2022). Contrastingly, *T*. *fortunei* shifted Q_10_ while the native species did not (Figs. 4 and 5). Similar acclimation of Q_10_ was found in other works focusing on evergreen species (e.g. Quan et al. 2020; Crous et al. 2022) and typically occurs when respiration is limited by low substrate availability (Atkin and Tjoelker 2003; van de Weg et al. 2013). The decrease in Q_10_ at high temperatures allowed the invasive palm to increase its A_net_ at warmer sites and could partially explain the broader T_80_ we observed at high temperatures (Supplementary Fig. S3). Q_10_ shifts have been associated with short-term changes of respiration with T_air_ (Atkin and Tjoelker 2003) and our findings contrast with studies measuring evergreen species in climatic chambers that observed no acclimation with temperature (e.g. Dusenge et al. 2020; Choury et al. 2022), potentially because Q_10_ also varies seasonally and its acclimation depends on the warming duration and intensity (Atkin et al. 2005; O’Sullivan et al., 2013). In fact, all our measured parameters (A_opt_, T_opt_, T_80_, R_25_, and Q_10_) shifted systematically with T_air_ within 2 wk in the 3 species. These results align with other studies (e.g. Kattge and Knorr 2007; Kumarathunge et al. 2019) stating that the commonly used 30-day acclimation period may be generally overestimated and that future works should consider using shorter acclimation periods (Smith and Dukes 2013; Reich et al. 2016; Vico et al. 2019). Second, all species (i.e. invasive and native ones) demonstrated a short acclimation duration and the invasive palm did not benefit from a more extensive photosynthetic and respiratory acclimation than native tree species.

### Impact of photosynthetic and respiratory acclimation on the leaf-level and plant C budget

Contrary to our expectations, the invasive palm generally showed lower C_leaf_ and C_tot_ than native species across all sites, even at the reference site where the palm invades the natural ecosystem (Fig. 6 and Supplementary Fig. S6). While higher C gains increase the competitiveness of plant species (Pearcy et al. 1987), lower C costs in invasive plants also play a significant role in invasion processes, and *T*. *fortunei* may have benefitted from other characteristics [e.g. longer photosynthetic period in autumn, lower whole-plant respiration, absence of herbivory, and slow tissue turnover (Fridley et al. 2022; Juillard et al. 2024)] to be invasive under current climatic conditions. At the reference site, *T*. *fortunei* also shares these characteristics with several other natives (e.g. *Ilex aquifolium*, *Hedera helix*) and non-native evergreen species (e.g. *Prunus laurocerasus*) currently spreading in the understory of sub-Mediterranean deciduous forests, which could confirm the important role of these characteristics in the forest composition changes (Conedera et al. 2018). *T*. *fortunei* currently benefits from a high propagule pressure in proximity to settlements (Conedera et al. 2018), yet C gain could become more important farther away in natural forests.

The similar C_leaf_ of *T*. *fortunei* between the sites (Fig. 6) can be explained by the relatively low increase in A_opt_ per °C T_opt_ (+0.11 *µ*mol m^2^ s^−1^ per °C T_opt_; Fig. 3), while T_opt_ showed the highest increase with T_air_ among the compared species (+0.60 °C per °C T_air_). Hence, accurate A_opt_ measurements not biased by stomatal closure at high VPD are crucial to predict whole tree C exchange acclimation realistically. At the leaf-level, the invasive palm performed similarly well at the reference site than in the 2 colder sites, while the native species had a lower C_leaf_ (Fig. 6), which shows that if *T*. *fortunei* is not damaged by frost in colder climates (Supplementary Figs. S4 and S5), its photosynthetic capacity allows to perform equally well as the native species. With global warming, extremes of cold conditions are becoming less frequent north of the Alps (Zubler et al. 2014), where *T*. *fortunei* is not yet as widely established as in the south. This trend is therefore likely to facilitate the spread of *T*. *fortunei* into colder climates beyond the sub-Mediterranean region, although further research is needed to confirm this. In contrast, native species acclimated more to the higher T_air_ at the Mediterranean site than the invasive palm (Fig. 6 and Supplementary Fig. S1), potentially limiting the capacity of the invasive palm to invade those environments. At the warmest site, C_leaf_ was lower in all species. Still, high temperatures especially limited the performance of *I*. *aquifolium* due to high respiratory rates (Figs. 6 and 7), highlighting the critical role of respiration on the C budget at temperatures above 30 °C.

Acclimation of photosynthesis and respiration allowed the invasive palm to increase C uptake and reduce C losses (leading to a higher C budget) in intermediate sites with milder conditions (Fig. 7). Nevertheless, the importance of acclimation was limited in extreme climates (both cold and hot ones), suggesting that shifts in photosynthetic and respiratory patterns are insufficient to compensate for the harsher climatic conditions. In contrast, acclimation allowed the native *T*. *cordata* to increase the C budget in the hottest environments (Fig. 7). Still, the highest C budget was observed in the Mediterranean climate (+2.5 °C), suggesting a limited enhancement of the C budget under global warming. Previous work also reported increased C gains in some temperate trees with a rise between +2 and +5 °C T_air_, especially because warming also leads to a certain extent to a longer growing season in deciduous trees (Duan et al. 2013; Grossiord et al. 2022). On the other hand, the absence of photosynthetic and respiratory acclimation benefits for *I. aquifolium* (Fig. 7) could be explained by the high respiratory rates that drastically reduced the C_leaf_ in all sites, even the cold ones (Fig. 6). As a shade-tolerant species, *I*. *aquifolium* tends to have high leaf *N* content, which has been associated with high respiration (Reich et al. 1998; Niinemets et al. 2003), potentially constraining the positive effect of T_air_ on its C budget.

Overall, photosynthetic and respiratory traits in *T*. *fortunei* remained similar in different climatic conditions, and our results suggest that thermal acclimation may not play an important role in the fast propagation of the invasive palm in recent decades. These results were obtained in an experimental setup, and their ecological relevance is limited by the design of the experiment and the fact that roots were bound in pots. Furthermore, the whole-plant C budget of *T*. *fortunei* was relatively difficult to calculate with the SPAC model due to a low signal-to-noise ratio (*R*^2^ = 0.17). Still, those findings align with reports including diverse experimental designs of invasive species conserving their initial niche (Verlinden and Nijs 2010; Liu et al. 2020; Ripley et al. 2020). In addition, our results confirm that some long-lived invasive species have not reached their potential range yet, as observed in Bradley et al. (2015). Their range expansion could, therefore, be predicted from physiological measurements directly but not from the extrapolation of their native or current range. Invasive plants often depend on many traits to colonize a new environment, and future invasion patterns in a warmer environment are difficult to predict. The majority of invasive species is fast growing and display high basal respiratory rates (Montesinos 2022), which could constrain their C budget under warming if they do not acclimate substantially. Our results show that *T*. *fortunei* does not follow such a resource acquisition strategy and instead displays low foliar respiratory rates. However, the large amount of living tissue in the palm stem is expected to drive an exponential increase in respiration with rising temperature (Schuler et al. 2025), which will likely affect its competitiveness in the future.

## Conclusion

Using a natural temperature gradient across European study sites, we showed that photosynthetic and respiratory acclimation occurs rapidly in young plants under understory conditions and can significantly affect the C budget of both invasive and native species. Contrary to expectations, the invasive palm *T*. *fortunei* did not exhibit greater acclimation capacity than its native competitors and showed a lower, though more stable, C budget overall. This species does not display the physiological traits typical of most invasive plants; however, it locally represents a broader group of non-native evergreens that likely compete through similar resource-acquisition strategies (Conedera et al. 2018). In central and southern Europe, summer T_air_ is projected to rise by 5 to 7 °C by the end of the century compared with 1995–2014 (SSP5-8.5) (Carvalho et al. 2021), which would reduce the C budget of all species examined in this study. It is therefore likely that both native sub-Mediterranean species and current invasive species in the region will be outcompeted by more thermophilic invaders in the future, driving shifts in forest species composition.

## Materials and methods

### Experimental design

**Figure 8.**
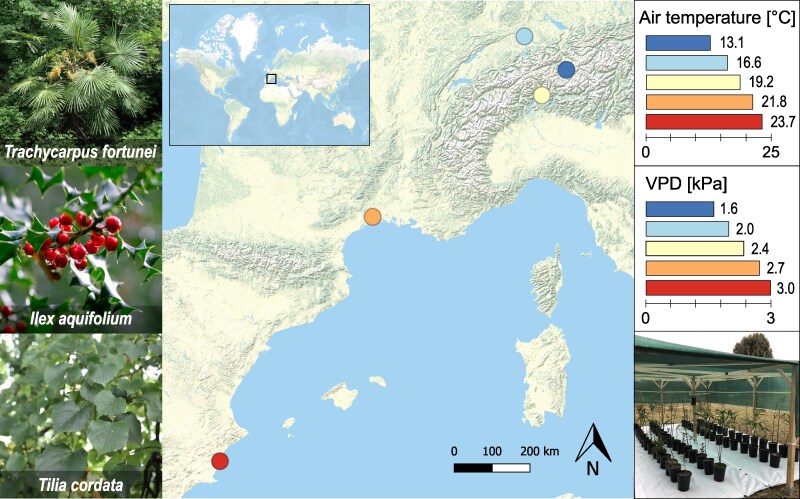
Map of the 5 experimental sites in Europe (from the coldest to the warmest: filisur (cold, dark blue), Birmensdorf (temperate, light blue), Cadenazzo (sub-Mediterranean, yellow), Montpellier (Mediterranean, orange), and Guardamar del Segura (semi-arid, red). On the left are pictures of the 3 focal species included in the study. The right panels show the mean air temperature and VPD within each shading infrastructure from April to October 2022 and 2023. At the bottom right is a picture showing the shading infrastructure.

We selected 5 sites across a temperature gradient in Europe for a transplant experiment: 1 reference site located in the sub-Mediterranean climate where the windmill palm (*Trachycarpus fortunei*) has one of the largest abundance relative to other species (i.e. the lowlands of the southern slope of the Swiss Alps; Fehr et al. 2020), and 4 sites with differences of respectively −6°, −3°, +3°, and +6 °C mean summer T_air_ (from April to October) compared with the reference site in the last 10 yr (2010–2020). This allowed us to assess the acclimation of the photosynthetic and respiration properties along a large T_air_ gradient, thereby covering regions where *T. fortunei* already has or could become invasive in future years (Fehr et al. 2023). In addition to the reference site in southern Switzerland (sub-Mediterranean climate), sites spanned from south-eastern Spain (thermic semi-arid), southern France (Mediterranean) to the Swiss Plateau (temperate), and the Swiss Alps (cold) (Fig. 8). The elevation of the sites ranged between 1 and 965 m a.s.l. (see Table 1 for more details). During the measurement period (2022 and 2023), mean annual T_air_ were 8.4, 13.3, 15.2, 17.7, and 21.8 °C from the coldest to the warmest site, respectively, with slightly warmer conditions in 2022 than in 2023 (Supplementary Fig. S6).

**Table 1.** Location of the experimental sites and associated climatic conditions

Experimental site	Filisur (CH)	Birmensdorf (CH)	Cadenazzo (CH)	Montpellier (FR)	Guardamar del Segura (ES)
Climate	Cold	Temperate	Sub-Mediterranean	Mediterranean	Semi-arid
Location	46°40′13.0″*N* 9°40′06.6″E	47°21′41.6″*N* 8°27′21.5″E	46°09′38.2″*N* 8°56′00.9″E	43°38′19.2″*N* 3°51′41.1″E	38°05′46.2″*N* 0°38′51.7″W
Altitude [m a.s.l.]	965	540	203	57	1
MAT 2010–2020 [°C]	8.4	12.0	12.3	15.8	18.7
T_air_ April-October 2010–2020 [°C]	12.6	16.5	17.7	20.1	22.5

MAT, mean annual temperature; T_air_, air temperature.

*T. fortunei* typically co-occurs with other species in the forest understory [e.g. the native evergreen European holly (*Ilex aquifolium*) and deciduous small-leaved linden (*Tilia cordata*)], which it occasionally excludes when dominating the canopy (Fehr et al. 2023). To simulate the natural half-shady understory environment, we constructed a wooden shading infrastructure (6 × 4 × 1.8 m) in an unobstructed area on all sites. The top and sides were covered with a permeable shading tissue obstructing 70% of sunlight. Mean yearly incoming solar radiation for 2022–2023 was similar among sites (Supplementary Fig. S6).

In November 2021, we dug out 200 saplings of *T. fortunei* of 50–70 cm in height in a freshly invaded sub-Mediterranean forest (Cugnasco, Switzerland; 46°10′15″ *N*, 8°55′49″ E, 205 m a.s.l., MAT 12.2 °C, MAP: 1,757 mm; 1.1 km from our reference site). We further bought 200 saplings of *T. cordata* (Morbio Superiore, Switzerland) and 150 saplings of *I. aquifolium* (Wiler bei Utzenstorf, Switzerland) from commercial producers. All individuals were immediately potted in 7 L pots with generic sandy forest soil made of 20% peat and mineral substrate (pH = 6.3; Ökohum; DE) and overwintered at the reference site.

In March 2022, 100 healthy individuals of each species (i.e. 300 saplings) were randomly separated into 5 groups of 20 individuals per species and transported to the 5 sites (*n* = 60 saplings per site). The plants for the coldest site were kept at the second coldest one (temperate climate) until April 2022 and from December 2022 to March 2023 to avoid exposure to temperatures below −15 °C as it could have killed *T. fortunei* (Fehr and Burga 2016). Despite this, 75% of the individuals of *T. fortunei* at the 2 coldest sites suffered frost damage during the winter of 2022–2023, limiting the number of replicates to 4 in 2023. At each site, pots were placed inside the shading infrastructure in 4 rows of 15 individuals each, alternating species to randomize potential differences in light and wind conditions. Each pot was individually connected to a drip irrigation system equipped with individual pressure resistances (Allenspach GreenTech AG, CH) to guarantee the same irrigation for each pot. Plants were watered automatically every 2 days at dawn to field capacity to ensure water availability throughout the experiment. We recorded T_air_, RH, and solar radiation (data logger: EM-100; air temperature and humidity: Atmos-14, Meter Group Inc.; USA; light quantum sensor: SQ-100X-SS, Apogee Instruments; USA) inside each shading infrastructure every 30 min at 1.5 m above the ground. We also monitored the soil moisture of each plant's pot at 10 cm depth monthly with a soil moisture meter (TDR-100, Spectrum Technologies; USA).

For 2 yr (2022–2023), the measurement campaigns were conducted in May, July, and September (6 in total) during 2 consecutive growing seasons (i.e. 2022 and 2023). We tracked leaf flush and senescence at each site with phenocams (IPX5, VisorTech; DE). To guarantee that we measured fully expanded leaves, the first campaign of each year in May was conducted 1 mo after all individuals of *T. cordata* had flushed.

### CO_2_ assimilation and respiration responses to air temperature

We measured the optimal temperature (T_opt_), net assimilation at the optimal temperature (A_opt_), thermal breadth (T_80_), dark respiration at 25 °C (R_25_), and respiration yield (Q_10_) of 1-yr-old (or fully developed of the current year in deciduous *T. cordata*), undamaged leaves of 10 individuals per species (4 individuals of *T. fortunei* in 2023). Hence, every year in May, 1 leaf per individual was selected for T_opt_, A_opt_, and T_80_ measurements, while an adjacent leaf was chosen for R_25_ and Q_10._

T_opt_, A_opt_, and T_80_ were obtained through CO_2_ assimilation response to T_air_ curves using portable photosynthesis systems (Li-6800, Licor Biosciences; USA) similar to Gauthey et al. (2023) and Deluigi et al. (2025). Measurements were conducted approximately every 1–2 h at 5 or 6 time points during the day, reflecting different ambient T_air_ from the sunrise (i.e. the coldest T_air_ of the day) to the middle of the afternoon (i.e. the warmest T_air_ of the day) to obtain the possible largest T_air_ range (from 12 to 35 °C on average across sites and campaigns). While diurnal effects cannot be fully excluded, the acclimation period and the use of C3 species under controlled conditions make it unlikely that our results were influenced by time-of-day variation (Rashid et al. 2020; Schuler et al. 2025). Air temperature within the Li-6800 chamber was set according to the ambient T_air_, which was measured continuously (RS-91, RS Instruments; DE). By doing so, we ensured to track the temperature response curve of the ambient T_air_ and to avoid possible artifacts associated with differences between the ambient temperature experienced by the plants and the conditions in the cuvette, as well as bias associated with the calculations of T_L_ within gas exchange systems (Still et al. 2019). To extend the temperature range, we additionally decreased and increased the temperature of the cuvette by 5 °C during the coldest and warmest time of the day, respectively. Relative humidity within the cuvette was increased at high temperatures to avoid stomatal closure due to high VPD. Hence, VPD ranged between 1 (fixed minimum) and maximum 3.5 kPa (when T_air_ reached > 38 °C). T_opt_, A_opt_, and T_80_ were measured at saturating light (PPFD of 1,500 *μ*mol m^−2^ s^−1^) and ambient CO_2_ (400 ppm). Measurements of R_25_ and Q_10_ were conducted as described above, except that the leaf of each individual was wrapped in aluminum foil for at least 30 min before each measurement to dark-acclimate the leaves and that light was reduced to 0 *μ*mol m^−2^ s^−1^ inside the cuvette during the measurements. All measurements were taken after the gas exchange rates had stabilized for at least 5 min.

We extracted T_opt_, A_opt_, and T_80_ of each individual by fitting our measurements of assimilation at different T_air_ with a parabolic curve in R (4.1.1, R Core Team, 2021), using the following equation as in Choury et al. (2022):


(1) $$A_{i}=A_{\mathrm{opt}}-b\;(T_{i}-T_{\mathrm{opt}}{)}^{2}$$

where *A_i_* is the net assimilation at temperature *T_i_* and *b* is the width of the curve. T_80_ was then calculated after resolving equation (1) for *A_i_* = 0.8 *A*_opt_ and isolating *T_i_*. Similarly, R_25_ and Q_10_ were obtained after fitting the respiration measurements at different *T*_air_ with an exponential curve in *R*, using the following equation (Choury et al. 2022):


(2) $$R_{i}=R_{25}\times \;Q_{10}^{\frac{\left(T_{i}-25\right)}{10}}$$

### SPAC modeling of leaf-level photosynthesis and respiration

To assess the temperature effect on the annual leaf-level C uptake (C_leaf_), we modeled the instantaneous leaf-level net photosynthesis at 30-min intervals from leaf flushing to senescence with a mechanistic SPAC model proposed by Garcia-Tejera et al. (2017) (Supplementary Fig. S7). The model was modified to account for the acclimation of temperature responses of photosynthesis and respiration based on leaf temperature (T_L_). The original model calculates iteratively the net assimilation (A_net_) and stomatal conductance (g_s_) based on environmental drivers (i.e. T_air_, RH, light availability) and physiological parameters (e.g. V_C,max,25_, (V_C,max_ at 25 °C), J_max,25_ (J_max_ at 25 °C), R_25_, Q_10_; list of all inputs in Supplementary Table S7). *A*_net_ is calculated as:


(3) $$A_{\mathrm{net}}=\;g_{s}\;(C_{a}-\;C_{i})\;+\;R_{d}$$

where *C_a_* is the ambient CO_2_ concentration, *C_i_* is the intracellular CO_2_ concentration, and *R_d_* is the dark respiration rate. *g_s_* is derived from its theoretical value at unlimited water availability (*g_s_*_,max_):


(4) $$g_{s,\max}=\;\frac{B\;(C_{i}-\Gamma )-\;R(DC_{i}+E)}{\left(E+F)(C_{i-\;}C_{a}\right)}$$

where *B* is the CO_2_ uptake limiting rate of either J_max_ or V_C,max_, Γ is the CO_2_ compensation point of photosynthesis, *E* is a metric of carboxylation and oxygenation rates, and *D* and *F* are constants. *g_s_* is then derived from *g_s_*_,max_ based on hydraulic parameters, water transport from the soil to the leaf, and transpiration (Supplementary Fig. S7, equations in Supplementary Table S8, detailed explanations in Supplementary Section S1).

To model T_L_, we used a leaf energy balance model by Kevin Tu (https://landfluxorg.godaddysites.com/tools) as in Marias et al. (2017). As such, T_L_ varied with T_air_, light, RH, and g_s_ following the equations from Jones (1992), Sridhar and Elliott (2002), and Monteith and Reifsnyder (2007) (Supplementary Table S9). As T_L_ impacts g_s_, and vice versa, we nested the SPAC optimization process for *g_s_* calculations into a second optimization process to calculate T_L_ (Supplementary Fig. S7). *R_d_* was calculated with T_L_ following equation (2). To incorporate thermal acclimation of photosynthesis in our model, V_C,max,25_ and J_max,25_ were adjusted for T_L_ following the peaked Arrhenius function equation as in Kumarathunge et al. (2019). We adjusted the entropy factor (ΔS, J mol^−1^ K^−1^) and the activation energy (Ha, kJ mol^−1^) in the peaked Arrhenius function with the general coefficients proposed by Kumarathunge et al. (2019) (Supplementary Tables S7 and S8). To consider the thermal acclimation of respiration, we adjusted R_25_ with T_air_ with linear species-specific equations that we derived from our results (Supplementary Table S7):


(6) $$R_{25}=\;0.50-0.0037\;T_{\mathrm{air}}\;\;T.fortunei\;$$

(7) $$R_{25}=\;1.42-0.0293\;T_{\mathrm{air}}\;I.aquifolium$$

(8) $$R_{25}=\;1.36-0.0317\;T_{\mathrm{air}}\;\;T.cordata$$

To parametrize the model, we measured once J_max,25_ and V_C,max,25_ on 5 individuals of the 3 species in July 2023 at our reference site with the Li-6800 following the methodology used in Didion-Gency et al. (2022) (Supplementary Section S2). In May 2022, we measured the minimum stomatal conductance (g_min_) on 4–5 saplings per species at EPFL (46°31′15.3″*N*, 6°34′04.0″E, Lausanne, CH) (Supplementary Section S3). In September each year (2022 and 2023), we measured the leaf area of 4–10 individuals of each species at every site. For this, we photographed 10 representative leaves of *I. aquifolium* and *T. cordata,* and all the leaves of *T. fortunei*. We extracted the leaf area using the software Fiji (Schindelin et al. 2012). The mean leaf area of *I. aquifolium* and *T. cordata* was multiplied by the total number of leaves of each individual to compute the total leaf area. Finally, the leaf root ratio (LRR) was obtained by dividing the total leaf area by the pot's upper surface.

We calibrated and validated the model with A_net_ measurements that we took diurnally at the 5 experimental sites during every campaign. A_net_ was measured approximately every hour with the Li-6800 under ambient light, T_air_, and RH. We randomly selected 70% of the measurements to calibrate influential model parameters that we could not measure similarly as in Grossiord et al. (2022) and Deluigi et al. (2025). The model was calibrated using a differential evolution (DEzs) Markov chain Monte Carlo (MCMC) sampler (Ter Braak and Vrugt 2008) using the R package BayesianTools (Hartig et al. 2023). For each species, we run 10,000 iterations of 3 independent chains. We confirmed the convergence of the calibration with the Gelman–Rubin diagnostic and a threshold of 1.1 (Gelman and Rubin 1992). We used the 30% remaining measurements to assess the goodness-of-fit (RMSE, percent bias, and Nash-Sutcliff-efficiency) between the measured and modeled A_net_ at each site. To estimate the effect of photosynthetic temperature acclimation on the plant C budget, we ran the model without acclimation by keeping V_C,max_, J_max_, R_25_, and Q_10_ constant (mean of the reference site).

### Statistical analyses

Species and site differences in T_opt_, T_80_, A_opt_, R_25_, and Q_10_ were tested through analysis of variance by using species (i.e. *T. fortunei*, *I. aquifolium*, and *T. cordata*) and climate (semi-arid, Mediterranean, sub-Mediterranean, temperate, and cold) and their interaction as fixed effects. No variable transformation was required to ensure homoscedasticity. Tukey's HSD post hoc tests were used to separately estimate differences between species or climates. Linear regressions were used to test the relationships between T_opt_, A_opt_, R_25_, Q_10,_ and T_air_ (mean of the 2 wk preceding the measurements) and between A_opt_ and T_opt_. All statistical tests were done using the software R (4.1.1, R Core Team, 2021).

## Supplementary Material

kiaf574_Supplementary_Data

## Data Availability

Data supporting this study are available on the Envidat Repository at https://doi.org/10.16904/envidat.712.

## Supplementary data

The following materials are available in the online version of this article.

**Supplementary Figure S1.** Optimal net assimilation (A_opt_), optimal temperature (T_opt_), thermal breathing (T_80_), respiration at 25 °C (R_25_), and respiration yield per 10 °C increase (Q_10_) (means ± S.e., *n* = 4–10 individuals per species) of all species during the 6 measurement campaigns in 2022–2023.

**Supplementary Figure S2.** Relationships between T_80_ (*n* = 4–10 individuals per species) averaged by campaigns and T_air_ of the 2 wk preceding the measurements for *T*. *fortunei*, *I*. *aquifolium*, and *T*. *cordata*.

**Supplementary Figure S3.** Thermal breath (T_80_) in function of the assimilation at the optimal temperature (A_opt_) and optimal temperature (T_opt_) for *T*. *fortunei*, *I*. *aquifolium*, and *T*. *cordata* during all campaigns in 2022 and 2023.

**Supplementary Figure S4.** Daily mean net C uptake of *T*. *fortunei*, *I*. *aquifolium*, and *T*. *cordata* at the 5 experimental sites from 15th October 2022 to 15th October 2023, multiplied by the leaf area.

**Supplementary Figure S5.** Leaf area (means ± Se, *n* = 4–10 individuals per species) of all species and sites in September 2023.

**Supplementary Figure S6.** Mean daily air temperature (top), vapor pressure deficit (VPD; middle), and photosynthetically active flux density (PPFD; bottom) within the shading infrastructures of the 5 experimental sites from March 2022 to October 2023.

**Supplementary Figure S7.** Simplified representation of the SPAC model.

**Supplementary Table S1.** Results of the 2-way ANOVA testing the effects of climate and species on the leaf-level optimal assimilation (A_opt_), temperature at the optimal assimilation (T_opt_), thermal breath of photosynthesis (T_80_), respiration rate at 25 °C (R_25_), and respiration yield (Q_10_).

**Supplementary Table S2.** Tukey's HSD post hoc test of optimal net assimilation (A_opt_), optimal temperature (T_opt_), thermal breathing (T_80_), dark respiration at 25 °C (R_25_), and respiration yield per 10 °C increase (Q_10_) (means ± S.e., *n* = 4–10 individuals per species) of all species and campaigns.

**Supplementary Table S3.** Squared-R (*R*^2^), percentage of bias (%Bias), and Nash-Sutcliff-efficiency (NSE) between measured and modeled data with and without acclimation for the 5 sites and the 3 species.

**Supplementary Table S4.** Results of the variance analyses of the modeled net assimilation in the SPAC model between the 5 climates and the 3 species.

**Supplementary Table S5.** Results of the Tukey's HSD test of the simulated yearly C budget between the reference and the other sites.

**Supplementary Table S6.** Results of the Tukey's HSD test of the simulated yearly C budget between the outputs of the model with and without acclimation.

**Supplementary Table S7.** List of the input parameters used in the SPAC model.

**Supplementary Table S8.** Variables, equations, and descriptions of the SPAC model.

**Supplementary Table S9.** Variables, equations, and descriptions of the leaf energy balance model.

**Supplementary Section S1.** Functioning of the SPAC model.

**Supplementary Section S2.** J_max,25_ and V_C,max,25_ measurements.

**Supplementary Section S3.** Minimum stomatal conductivity measurements.

**Supplementary Section S4.** Tree growth, soil humidity, and chlorophyll content.
