# Supplementary material for: Thermal acclimation fails to confer a carbon budget advantage to invasive species over natives
Source: Plant Physiol. 2025 Nov 10;199(3):kiaf574. doi: 10.1093/plphys/kiaf574 (PMC12631787; doi:10.1093/plphys/kiaf574)
Supplement: kiaf574_Supplementary_Data [file kiaf574_supplementary_data.pdf]

Supplementary data

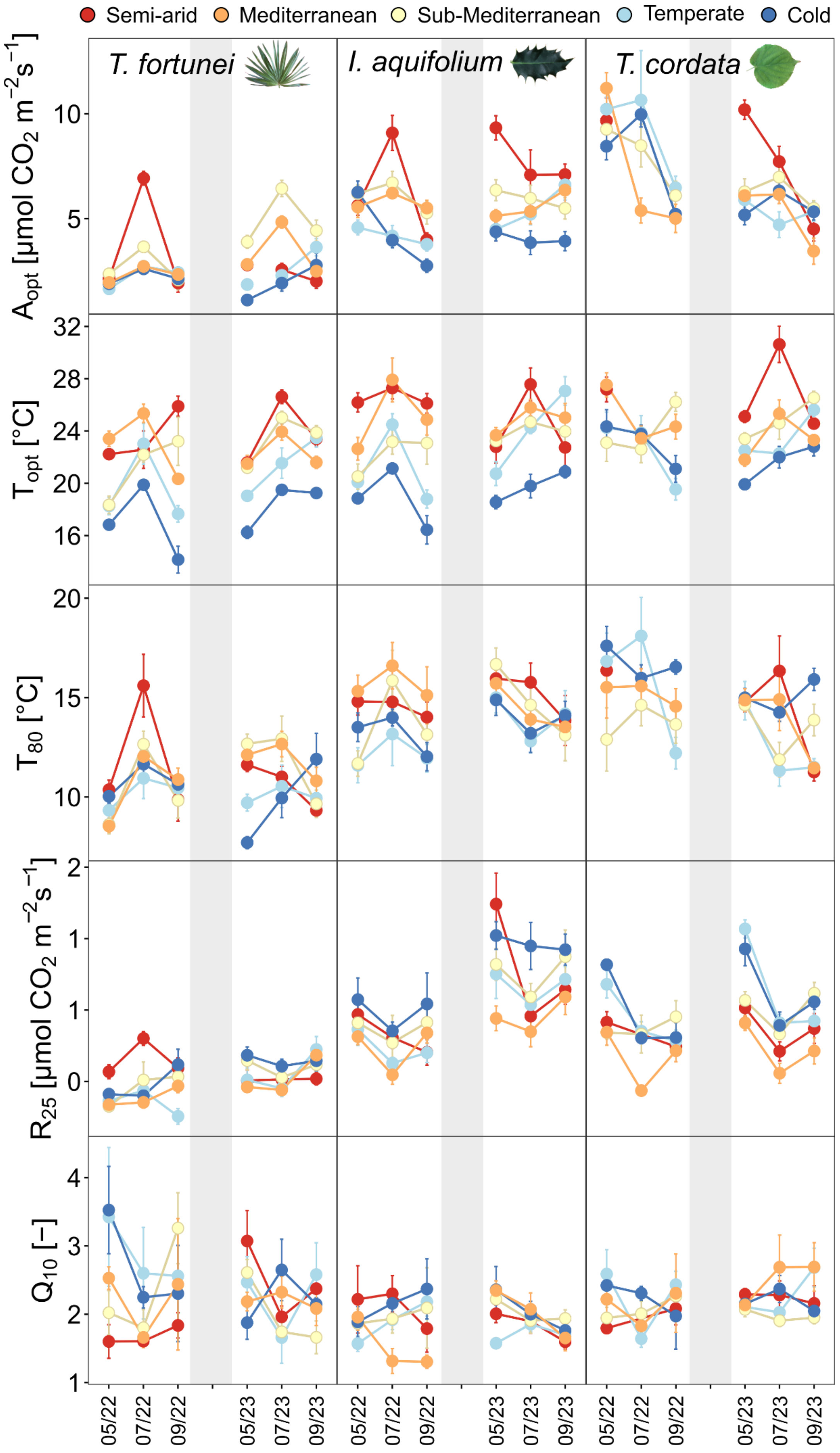
Semi-arid
Mediterranean
Sub-Mediterranean
Temperate
Cold
T. fortunei
I. aquifolium
T. cordata
A_{opt} [µmol CO_2 $m^{-2}s^{-1}$]
T_{opt} [°C]
T_{80} [°C]
R_{25} [µmol CO_2 $m^{-2}s^{-1}$]
Q_{10} [–]
05/22
07/22
09/22
05/23
07/23
09/23

Supplementary Figure S1: Optimal net assimilation (A_{opt}), optimal temperature (T_{opt}), thermal breathing (T_{80}), respiration at 25°C (R_{25}), and respiration yield per 10°C increase (Q_{10}) (means ± s.e., n = 4-10 individuals per species) of all species during the six measurement campaigns in 2022-2023. Significant differences between species (Tukey's HSD post hoc test, alpha = 0.05) at each campaign are indicated with different letters in Supplementary Table S2.

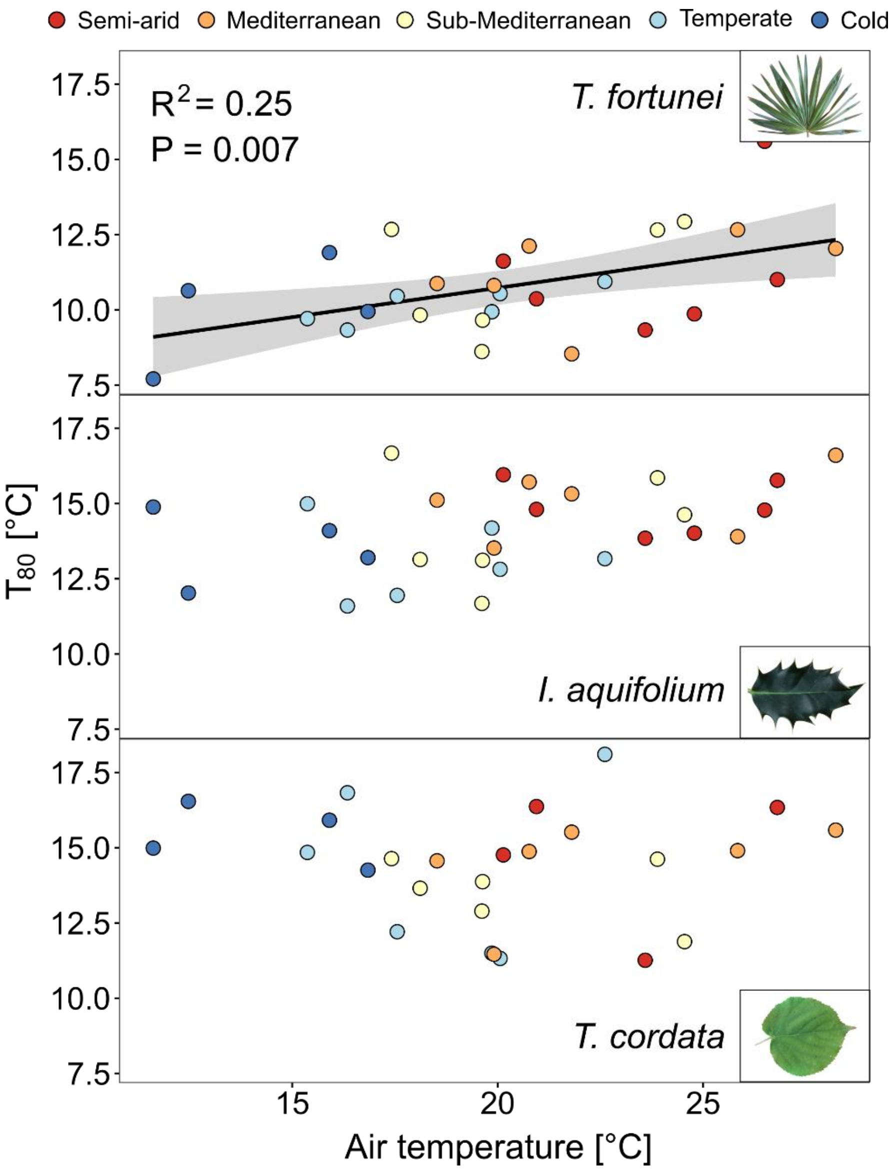

8 **Supplementary Figure S2:** Relationships between T_{80} (n = 4-10 individuals per species)
9 averaged by campaigns and T_{air} of the two weeks preceding the measurements for *T. fortunei*,
10 *I. aquifolium*, and *T. cordata*. Colors represent climates from blue to red, going from the coldest
11 to the warmest. The regression lines (ordinary least squares) were fitted with a linear model
12 when significant.

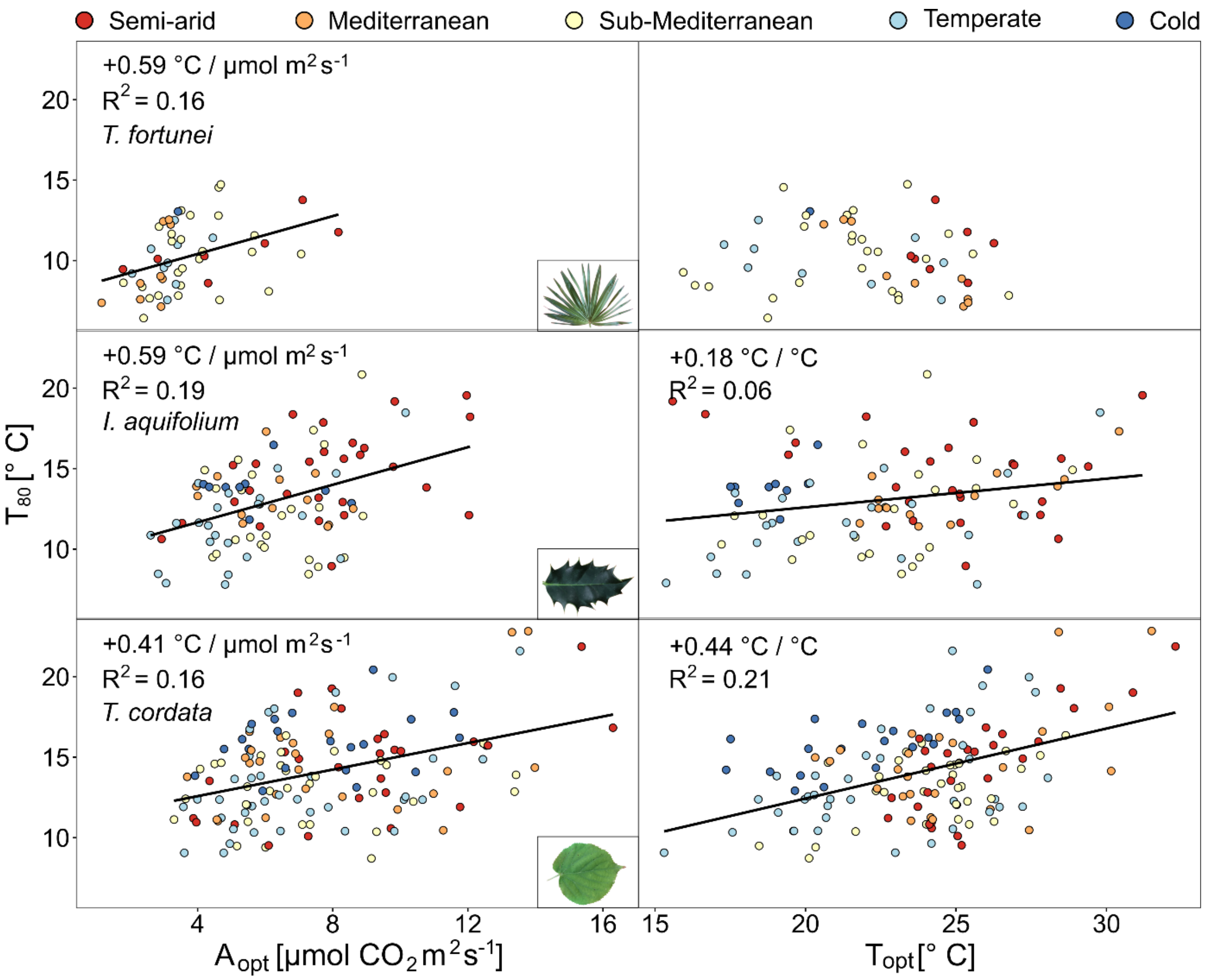

Supplementary Figure S3: Thermal breath (T_{80}) in function of the assimilation at the optimal temperature (A_{opt}) and optimal temperature (T_{opt}) for *T. fortunei*, *I. aquifolium*, and *T. cordata* during all campaigns in 2022 and 2023. Colors represent sites from blue to red, going from the coldest to the warmest. The regression lines (ordinary least squares) were fitted with a linear model when significant.

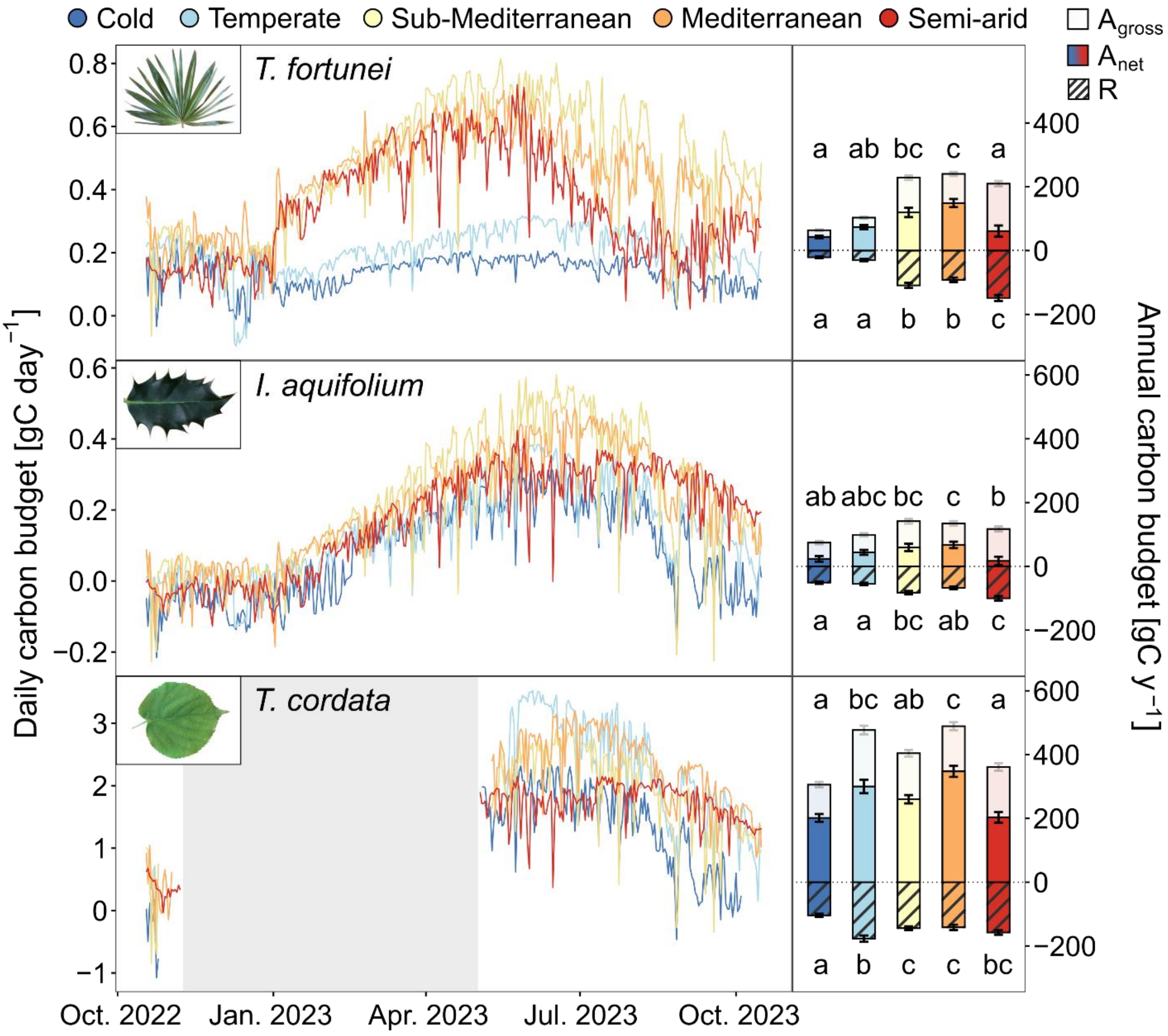

Supplementary Figure S4: Daily mean net C uptake of *T. fortunei*, *I. aquifolium*, and *T. cordata* at the five experimental sites from 15th October 2022 to 15th October 2023, multiplied by the leaf area. The right panels show the yearly C uptake at each site. Gross assimilation (A_{gross}) corresponds to the total length of the bars above 0, net assimilation (A_{net}) corresponds to the plain bars, whereas respiration (R) bars are dashed in black. Standard error bars indicate the uncertainty of $J_{max,25}$, $V_{Cmax,25}$, R_{25}, and Q_{10} modeled (n = 37-57). Different letters indicate significant differences ($p < 0.05$) between the sites based on Tukey's HSD test.

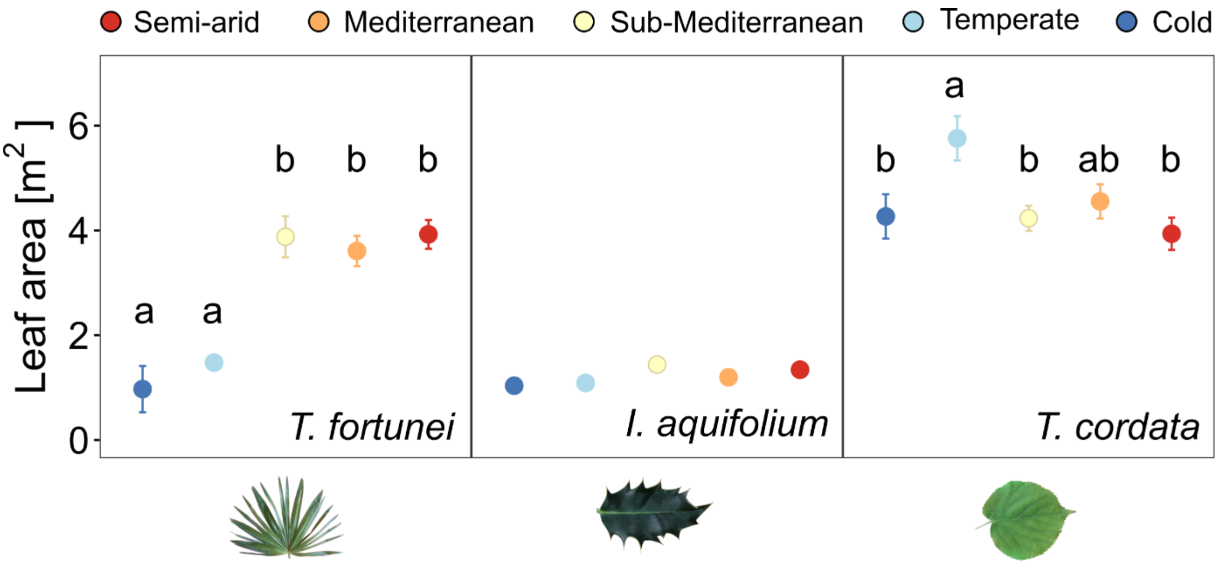

Supplementary Figure S5: Leaf area (means +/- s.e., n = 4-10 individuals per species) of all species and sites in September 2023. Different letters indicate significant differences (p < 0.05) between the sites based on Tukey's HSD test.

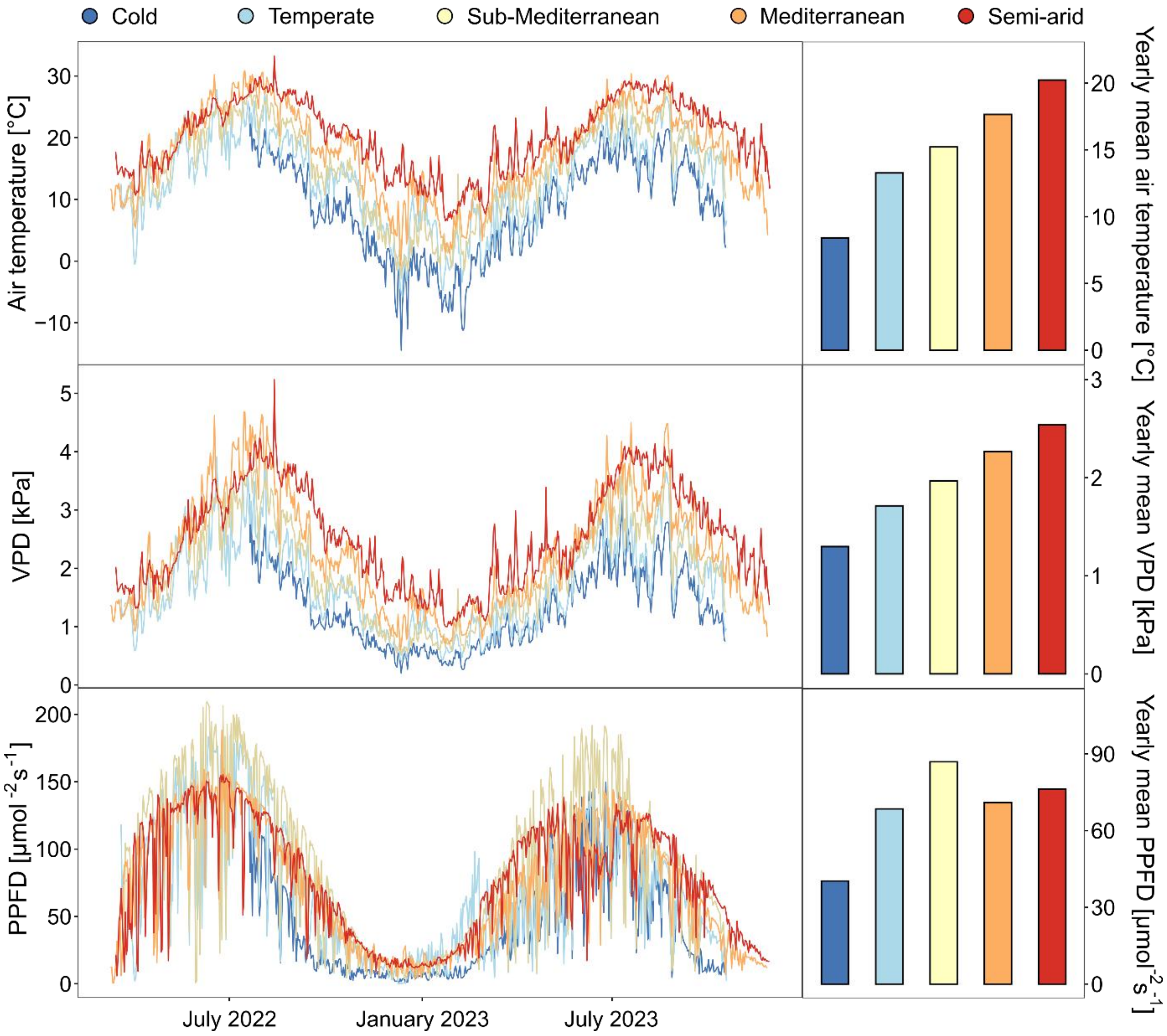

Supplementary Figure S6: Mean daily air temperature (top), vapor pressure deficit (VPD; middle), and photosynthetically active flux density (PPFD; bottom) within the shading infrastructures of the five experimental sites from March 2022 to October 2023. The yearly mean values of the three variables are presented on the right panel by sites.

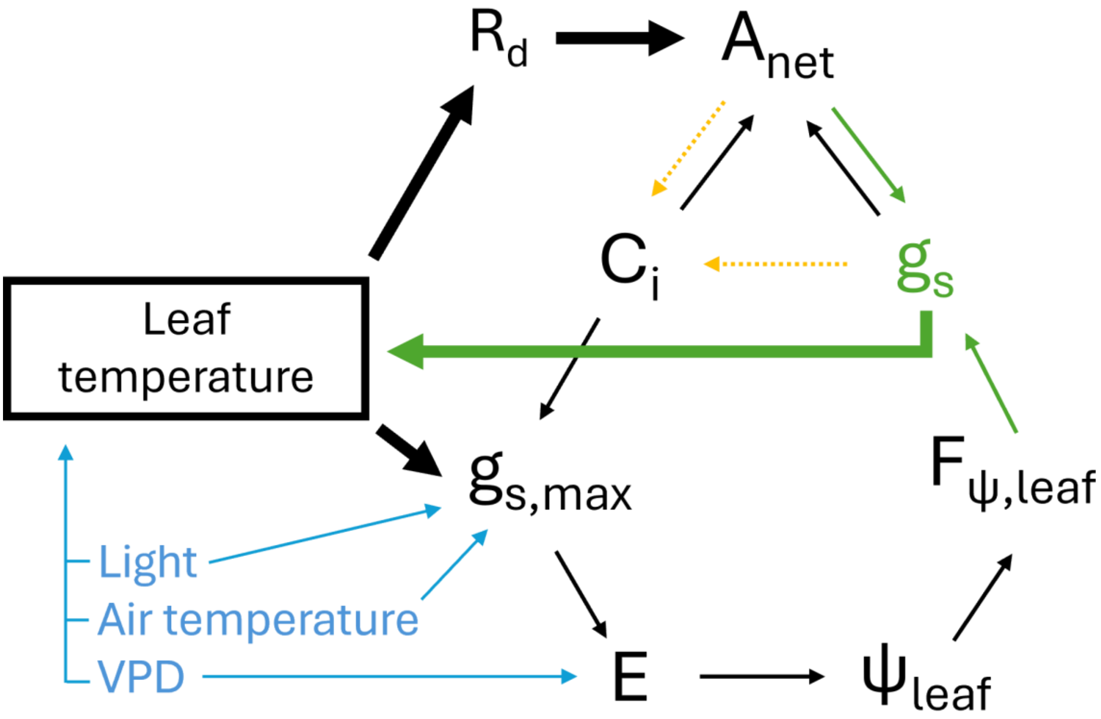

Supplementary Figure S7: Simplified representation of the SPAC model. The optimization procedure to find a stable stomatal conductance (g_s, thin arrows) is nested within the optimization procedure to find a stable leaf temperature (thick arrows). Colors are used to show different steps of the process. (1) Leaf temperature is calculated from environmental drivers (in blue) with an energy balance module. (2) Initial values of intercellular CO_2 concentration (C_i) and g_s are used to initiate the optimization procedure on g_s. (3) Iteratively, g_s is optimized from the net photosynthesis rate (A_{net}) and a correction factor ($F_{\psi,leaf}$) (in thin green) after computing the maximal stomatal conductance ($g_{s,max}$), evaporation (E), and leaf water potential (ψ_{leaf}). The iterative process stops when the new C_i, calculated with the optimized g_s (in yellow dotted), matches the Ci of the previous iteration. (5) Optimized g_s is used to calculate a new leaf temperature compared with the initial leaf temperature (thick green arrow). New iterations on both loops are run as long as values differ.

Supplementary Table S1: Results of the two-way ANOVA testing the effects of climate and species on the leaf-level optimal assimilation (A_{opt}), temperature at the optimal assimilation (T_{opt}), thermal breath of photosynthesis (T_{80}), respiration rate at 25°C (R_{25}), and respiration yield (Q_{10}). Significant effects ($p < 0.05$) are shown in bold.

**Variable**	**Fixed effect**	**Df**	**Sum Sq**	**Mean Sq**	**F-value**	**P-values**
A_{opt}	Climate	4	192.2	48	11.7	**< 0.001**
	Species	2	1972.7	986.4	239.3	**< 0.001**
	Climate:Species	8	111	13.9	3.4	**< 0.001**
	Residuals	652	2687	4.1		
T_{opt}	Climate	4	1991	497.7	59.9	**< 0.001**
	Species	2	972	485.9	58.5	**< 0.001**
	Climate:Species	8	158	19.8	2.4	**0.0158**
	Residuals	652	5418	8.3		
T_{80}	Climate	4	97	24.1	3.3	**0.0112**
	Species	2	1859	929.4	126.4	**< 0.001**
	Climate:Species	8	157	19.7	2.7	**0.0068**
	Residuals	652	4795	7.4		
R_{25}	Climate	4	4.4	1.1	17.3	**< 0.001**
	Species	2	23.9	11.9	186.9	**< 0.001**
	Climate:Species	8	2.0	0.3	3.9	**< 0.001**
	Residuals	682	43.6	0.1		
Q_{10}	Climate	4	128	31.9	2.6	**0.0352**
	Species	2	141	70.5	5.7	**0.0034**
	Climate:Species	8	411	51.4	4.2	**< 0.001**
	Residuals	682	8378	12.3		

Supplementary Table S2: Tukey's HSD post hoc test of optimal net assimilation (A_{opt}), optimal temperature (T_{opt}), thermal breathing (T_{80}), dark respiration at 25°C (R_{25}), and respiration yield per 10°C increase (Q_{10}) (means ± s.e., n = 4-10 individuals per species) of all species and campaigns. Significant differences between sites for each species and campaign are indicated with different letters ($p < 0.05$) .

Campaign	Species	Climate	A_{opt}	T_{opt}	T_{80}	R_{25}	Q_{10}	Campaign	Species	Climate	A_{opt}	T_{opt}	T_{80}	R_{25}	Q_{10}
2023 May	*T. fortunei*	S.-A.	-	b	b	b	-	2023 May	*T. fortunei*	S.-A.	a	b	b	b	-
		Med.	-	b	a	a	-			Med.	a	b	b	-	b
		Sub-Med.	-	a	a	a	-			Sub-Med.	c	b	b	-	ab
		Temp.	-	a	ab	a	-			Temp.	ab	a	a	-	a
		Cold	-	a	ab	ab	-			Cold	b	c	c	-	ab
	I. aquifolium	S.-A.	-	c	b	-	-		*I. aquifolium*	S.-A.	c	a	-	-	-
		Med.	-	a	b	-	-			Med.	ab	a	-	b	-
		Sub-Med.	-	ab	a	-	-			Sub-Med.	b	a	-	ab	-
		Temp.	-	ab	a	-	-			Temp.	a	ab	-	b	-
		Cold	-	b	ab	-	-			Cold	a	b	-	ab	-
	T. cordata	S.-A.	-	-	-	ac	-		*T. cordata*	S.-A.	b	b	-	a	-
		Med.	-	-	-	c	-			Med.	a	ac	-	a	-
		Sub-Med.	-	-	-	c	-			Sub-Med.	a	ab	-	a	-
		Temp.	-	-	-	ab	-			Temp.	a	a	-	b	-
		Cold	-	-	-	b	-			Cold	a	c	-	b	-
2023 July	*T. fortunei*	S.-A.	b	ab	b	b	-	2023 July	*T. fortunei*	S.-A.	a	d	-	b	-
		Med.	a	a	ab	a	-			Med.	c	ac	-	-	-
		Sub-Med.	a	ab	ab	ab	-			Sub-Med.	b	cd	-	-	-
		Temp.	a	ab	a	a	-			Temp.	a	ab	-	-	-
		Cold	a	b	ab	a	-			Cold	a	b	-	-	-
	I. aquifolium	S.-A.	c	b	-	-	b		*I. aquifolium*	S.-A.	a	b	-	ab	-
		Med.	ab	b	-	-	a			Med.	ab	ab	-	b	-
		Sub-Med.	b	a	-	-	ab			Sub-Med.	ab	ab	-	ab	-
		Temp.	a	ab	-	-	ab			Temp.	ab	a	-	a	-
		Cold	a	a	-	-	b			Cold	b	c	-	ab	-
	T. cordata	S.-A.							*T. cordata*	S.-A.	b	b	b	a	-
		Med.	b	-	-	b	ab			Med.	ab	a	ab	a	-
		Sub-Med.	ab	-	-	a	ab			Sub-Med.	ab	a	a	a	-
		Temp.	a	-	-	a	a			Temp.	a	a	a	ab	-
		Cold	a	-	-	a	b			Cold	ab	a	ab	ab	-
2023 Sept	*T. fortunei*	S.-A.	-	c	-	-	-	2023 Sept	*T. fortunei*	S.-A.	b	ab	-	b	-
		Med.	-	ab	-	b	-			Med.	b	bc	-	-	-
		Sub-Med.	-	bc	-	a	-			Sub-Med.	a	a	-	-	-
		Temp.	-	a	-	b	-			Temp.	ab	ab	-	-	-
		Cold	-	d	-	b	-			Cold	ab	c	-	-	-
	I. aquifolium	S.-A.	ab	b	-	-	-		*I. aquifolium*	S.-A.	a	b	-	-	ab
		Med.	b	b	-	-	-			Med.	ab	ab	-	-	-
		Sub-Med.	b	b	-	-	-			Sub-Med.	ab	ab	-	-	-
		Temp.	a	a	-	-	-			Temp.	a	a	-	-	-
		Cold	a	a	-	-	-			Cold	b	b	-	-	-
	T. cordata	S.-A.							*T. cordata*	S.-A.	-	abc	a	-	-
		Med.	-	bc	ab	-	-			Med.	-	bc	ab	ab	-
		Sub-Med.	-	b	ab	-	-			Sub-Med.	-	a	bc	ab	-
		Temp.	-	a	a	-	-			Temp.	-	ab	a	ab	-
		Cold	-	ac	b	-	-			Cold	-	c	c	a	-

Supplementary Table S3: Squared-R (R^2), percentage of bias (%Bias), and Nash-Sutcliff-efficiency (NSE) between measured and modeled data with and without acclimation for the five sites and the three species.

	**Acclimation in model**			**No acclimation in model**		
Species	R^2	%Bias	NSE	R^2	%Bias	NSE
T. fortunei	0.17	-0.69	0.11	0.15	-0.83	0.09
I. aquifolium	0.61	1.41	0.47	0.61	1.54	0.42
T. cordata	0.66	1.72	0.62	0.69	2.57	0.62
ALL	0.61	0.91	0.54	0.61	1.23	0.53

Supplementary Table S4: Results of the variance analyses of the modeled net assimilation in the SPAC model between the five climates and the three species. The left column corresponds to the outputs of the model with air temperature and acclimated physiology (*i.e.*, based on our measurements on each site). In contrast, the right column presents results obtained with non-acclimated physiological traits (*i.e.*, the traits from the reference site) to isolate the effect of the air temperature. Significant effects ($p < 0.05$) are shown in bold.

	**ΔT and Acclimation**			**ΔT only**		
	DF	F-value	P-value	DF	F-value	P-value
Climate	4	11.261	**<0.001**	4	4.331	**0.002**
Species	2	23.721	**<0.001**	2	17.398	**< 0.001**
Climate:Species	8	2.372	**0.016**	8	0.863	0.547
Residuals	682			670		

Supplementary Table S5: Results of the Tukey's HSD test of the simulated yearly C budget between the reference and the other sites. The left column corresponds to the outputs of the model with air temperature and acclimated physiology (i.e., based on our measurements on each site), while the right column presents results obtained with non-acclimated physiological traits (i.e., the traits from the reference site) to isolate the effect of the air temperature. Significant effects ($p < 0.05$) are shown in bold.

		**ΔT and Acclimation**	**ΔT only**
Species	Relation	*P*-value	*P*-value
T. fortunei	SubMed - Cold	0.649224	0.999994
	SubMed -Tem	0.073901	0.991281
	SubMed - Med	0.262958	0.975158
	SubMed - SA	**0.04093**	0.946181
I. aquifolium	SubMed - Cold	0.483926	0.402412
	SubMed - Tem	0.999945	0.999971
	SubMed - Med	0.733006	0.999938
	SubMed - SA	0.11118	0.553063
T. cordata	SubMed - Cold	**0.040258**	**0.012745**
	SubMed - Tem	0.318164	0.891027
	SubMed - Med	**0.023818**	0.056745
	SubMed - SA	0.760221	**0.000103**

Supplementary Table S6: Results of the Tukey's HSD test of the simulated yearly C budget between the outputs of the model with and without acclimation (*i.e.*, based on our measurements at each site or with the physiologic traits of the reference site, respectively). The variance corresponds to the uncertainty of $J_{max,25}$, and $V_{Cmax,25}$, R_{25}, and Q_{10} measured (n = 37-57). Significant effects (p < 0.05) are shown in bold.

**ΔT & Acclimation *vs.* ΔT only**		
Species	Climate	*P*-value
T. fortunei	Cold	0.086
	Temperate	**0.015**
	Sub-Mediterranean	0.572
	Mediterranean	**0.046**
	Semi-Arid	0.211
I. aquifolium	Cold	0.480
	Temperate	0.498
	Sub-Mediterranean	0.526
	Mediterranean	0.054
	Semi-Arid	0.758
T. cordata	Cold	0.163
	Temperate	0.652
	Sub-Mediterranean	0.563
	Mediterranean	**<0.001**
	Semi-Arid	**0.005**

Supplementary Table S7: List of the input parameters used in the SPAC model.

Variable	Symbol	Unit	Value			Source
Environmental parameters						
Air temperature	Ta	°C				Measured
Air humidity	RH	%				Measured
Light availability	L	PPFD				Measured
Wind speed	WS	m s^{-1}	2			-
Ambient CO2	Ca	µmol mol^{-1}	400			-
Photosynthetic parameters			*T. fortunei*	*I .aquifolium*	*T .cordata*	
Max. catalytic activity of Rubisco at 25 °C	vcmax	µmol m^{-2} s^{-1}	25.97	33.48	32.18	Measured
Max. ratio of electron transport at 25 °C	jmax	µmol m^{-2} s^{-1}	62.72	84.11	61.90	Measured
Respiration at 25 °C	Rd_25	µmol m^{-2} s^{-1}	0.41	0.85	0.75	Measured
Respiration yield	Q_{10}	-	2.297838	1.934928	2.182466	Measured
Leaf root ratio	LRR	m^2 m^{-2}	1.907561	0.709583	3.173085	Measured
Fraction of sunlit leaves	f_leaf_sun	-	1			-
Reference leaf water potential	Psi_f	kPa	-462	-445	-470	Calibrated
Shape factor	s_f	kPa^{-1}	0.0061	0.0061	0.0980	Calibrated
Minimum stomatal conductance	g_{min}	µmol m^{-2} s^{-1}	0.0038	0.0028	0.0060	Measured
Proportion factor	m	-	0.02	0.17	0.3	Calibrated
Degree of curvature PAR / J_{max}	gamma	µmol m^{-2}	0.9			Buckley et al. (2014) Garcia-Tejera (2023)
Hydrological parameters						
Soil water potential	Psi_soil	kPa	-300			Garcia-Tejera (2023)
Root resistance	R_root	kPa m^2 s kg^{-1}	10000			Grossiord (2022)
Xylem resistance	R_xylem	kPa m^2 s kg^{-1}	0.0625	0.0766	0.0499	Grossiord (2022)
Soil resistance	R_soil	kPa	100000			Garcia-Tejera (2023)
Leaf temperature						
Leaf angle from horizontal	i	°	30			Leaf Energy Balance function (http://landflux.org/Tools.php)
Absorptance to SWR	a_{SWR}	%	0.5			LEB function
Emissivity	em	-	0.97			LEB function
Characteristic dimension	d	mm	320	40	60	Measured
Shape of the leaf	shape_index	Cat.	1	2	1	Measured
Photosynthetic parameters for thermal acclimation						
Activation energy J_{max}	Ha	kJ mol^{-1}	51.5			Kumarathunge et al. (2019)
Activation energy V_{Cmax}			79.4			Kumarathunge et al. (2019)
Deactivation energy	Hd	kJ mol^{-1}	200			Kumarathunge et al. (2019)
Entropy factor J_{max}	Delta_S	$°C^{-1}$	662.3			Kumarathunge et al. (2019)
Entropy factor V_{Cmax}			647.9			Kumarathunge et al. (2019)
Respiration parameters for thermal acclimation						
Slope for R_{25} acclimation		µmol m^{-2} s^{-1} / °C	-0.0037	-0.0293	-0.0317	Measured
Intercept for R_{25} acclimation		µmol m^{-2} s^{-1}	0.50	1.42	1.36	Measured

Supplementary Table S8: Variables, equations, and descriptions of the SPAC model

Var.	Symbol	Unit	Equation	Definition of var. in the equation
Optimization process over T_L				
Dark respiration	R	μmol m^2 s^{-1}	$R_{25} * Q_{10}^{TL-25 / 10}$	R_{25} = Respiration at 25°C; Q_{10} = Respiration yield; T_L = leaf temperature
Max. catalytic activity of Rubisco at 25 °C	V_{Cmax}	μmol m^{-2} s^{-1}	Equation (5) in the main manuscript	
Max. ratio of electron transport at 25 °C	J_{max}	μmol m^{-2} s^{-1}	Equation (5) in the main manuscript	
Activation energy for V_{Cmax}	H_a	kJ mol^{-1}	39.7 + 1.14 T_{growth}	T_{growth} = mean air temperature of the two previous weeks
Activation energy for J_{max}	H_a	kJ mol^{-1}	27.2 + 0.26 T_{growth}	
Entropy factor for V_{Cmax}	ΔS	J mol^{-1} K^{-1}	645.1 – 0.38 T_{growth}	
Entropy factor for J_{max}	ΔS	J mol^{-1} K^{-1}	653.9 – 0.85 T_{growth}	
Optimization process over g_s				
Maximal stomatal conductance	$g_{s,max}$	μmol m^{-2} s^{-1}	Minimum of [B (C_i - Γ) - R (EC_i + D)] / [((EC_i + D)(C_i - C_a)] for VC_{max} or J, see Garcia *et al.*, (2017) for details	C_i = Internal CO_2 concentration [μmol m^{-1}]; C_a = ambient CO_2 concentration [μmol m^{-1}]; Γ = CO_2 compensation point [μmol CO_2 m^{-1}]; B = VC_{max} or J (calculated from Jmax, quantum efficiency, and APAR) D = 210.1 or 0.8 (combination of Michaelis–Menten coefficients and Γ) E = 1 or 4
Gross assimilation	A_{gross}	μmol m^{-2} s^{-1}	g_s (C_a- C_i) + R	C_a = ambient CO_2 concentration [μmol m^{-1}]
Transpiration	TR	Kg s^{-1} m^{-2}	$g_{s,max}$ LRR VPD / P	LRR = Leaf root ratio [m^2 leaf m^{-2} soil]; VPD = water vapor pressure deficit of the air [kPa]; P = atmospheric pressure [Pa]
Leaf water potential	ψleaf	kPa	($ψ_{soil}$ / (R_{soil} + R_{root})) / (1 / (R_{soil} + R_{root})) – TR * [(R_{soil} + R_{root} + R_{xylem}) / $f_{leaf\ sun}$]	$ψ_{soil}$ = ; R_{soil}, R_{root}, R_{xylem} = soil, root, and xylem hydraulic resistance [s m kPa kg^{-1}]; $f_{leaf\ sun}$ = fraction of the leaves illuminated
Modifying factor of leaf water potential	$f_{ψ,leaf}$	-	(1+ $e^{sf\ ψf}$) / (1+ $e^{sf\ (ψf – ψleaf)}$)	Sf = Shape factor [kPa^{-1}]; ψf = Reference leaf water potential [kPa]
Stomatal conductance	g_s	μmol m^{-2} s^{-1}	g_{min} + [$f_{ψ,leaf}$ m A_{gross} / (C_i - Γ)]	g_{min} = minimal stomatal conductance [μmol m^{-2} s^{-1}] ; m = proportionS.-A.ty factor between photosynthesis and stomatal conductance [-]; C_i = Internal CO_2 concentration [μmol m^{-1}]; Γ = CO_2 compensation point [μmol CO_2 m^{-1}]

Supplementary Table S9: Variables, equations, and descriptions of the leaf energy balance model

Var.	Symbol	Unit	Equation	Definition of var. in the equation
e_{sat}	Saturation vapor pressure	kPa	$a\ e^{b\ Tair\ /\ (Tair\ +\ z)}$	a = 0.61121 [kPa]<br>b = 17.502 [-]<br>z = 240.97 [°C]<br>Tair = air temperature [°C]
e_a	Water vapor pressure of the air	kPa	e_{sat} (RH / 100)	RH = relative humidity [%]
s	Slope of e_{sat} / T curve	kPa °C^{-1}	$e_{sat}\ b\ z/((T_{air} + z)^2)$	b = 17.502 [-]<br>z = 240.97 [°C]<br>Tair = air temperature [°C]
VPD	Water vapor pressure deficit of the air	kPa	$e_{sat} - e_a$	
SWR_{abs}	Absorbed short-wave radiation	W m^{-2}	a_{SWR} cos(i) SWR	a_{SWR} = absorptance to SWR [%]<br>i = inclination of the leaf from horizontal [°]<br>SWR = short-wave radiation [W m^{-2}]
LWR_{in}	Incoming long-wave radiation	W m^{-2}	$1.31(10\ e_a / T_{air})^{(1/7)}$ SB $(T_{air} + 273.15)^4$	Tair = air temperature<br>SB = Stefan-Boltzman constant = 5.67e-8 [W m^{-2} K^{-4}]
$LWR_{out,i}$	Isothermal outgoing long-wave radiation	W m^{-2}	em SB $(T_{air} + 273.15)^4$	em = emissivity = 0.97 [-]<br>SB = Stefan-Boltzman constant = 5.67e-8 [W m^{-2} K^{-4}]
Rni	Isothermal net radiation	W m^{-2}	$SWR_{abs} + LWR_{in} - LWR_{out,i}$	
r_r	Radiative resistance	s m^{-1}	P C_p / (4 em SB (Tair + 273.15)^3)	P = density of air = 1.292 [kg m^{-3}]<br>Cp = heat capacity of dry air = 1010 [J kg^{-1} K^{-1}]<br>em = emissivity = 0.97 [-]<br>SB = Stefan-Boltzman constant = 5.67e^{-8} W m^{-2} K^{-4}
r_{bl}	Leaf boundary-layer resistance	s m^{-1}	$1 / (g_x (WS^{jx} / d^{1-jx})$	Gx [m] & Jx [-] = coefficients depending on leaf shape (flat = 0.00662 & 0.5; cylinder = 0.00403 & 0.6; sphere = 0.00571 & 0.6)<br>WS = wind speed [m s^{-1}]<br>d = characteristic dimension [mm]
r_{blr}	Boundary-layer + radiative resistance	s m^{-1}	$1 / (r_{bl}^{-1} + r_r^{-1})$	
y_m	Modified psychrometric constant	kPa K^{-1}	$y\ (r_{st} / r_{blr})$	y = psychrometric constant = 0.066 [kPa K^{-1}]
T_{leaf}	Leaf temperature	°C	$T_{air} + (y_m\ R_{ni}\ r_{blr} / (p\ C_p) - VPD) / (s + y_m)$	P = density of air = 1.292 [kg m^{-3}]<br>Cp = heat capacity of dry air = 1010 [J kg^{-1} K^{-1}]

Supplementary Section S1: Functioning of the SPAC model

The model consists of finding by iteration an equilibrium for leaf temperature (T_L) as well as intracellular CO_2 concentration (C_i) and stomatal conductance (g_S). Along with physiological and environmental variables (Supplementary Table S7), an initial value for C_i and g_s has to be provided for the first iteration. First, T_L is calculated from the leaf energy balance function and varies, in particular, with T_{air} and g_s (equations shown in Supplementary Tables S8 and S9). T_L will not be calculated again before a stable g_s is iteratively computed in the model.

A_{net} is calculated as:

$$A_{net} = g_s\,(C_a - C_i) + R_d \qquad \text{(S1)}$$

where C_a is the ambient CO_2 concentration, C_i is the intracellular CO_2 concentration, and R_d is the dark respiration rate. R_d is calculated from:

$$R_d = R_{25} * Q_{10}{}^{TL-25\,/\,10}$$

where R_{25} and Q_{10} were interpolated from our gas exchange measures and acclimated depending on the mean air temperature of the two previous weeks:

$$R_{25} = 0.50 - 0.0037\,T_{air} \qquad \textit{T. fortunei} \qquad \text{(S2)}$$

$$R_{25} = 1.42 - 0.0293\,T_{air} \qquad \textit{I. aquifolium} \qquad \text{(S3)}$$

$$R_{25} = 1.36 - 0.0317\,T_{air} \qquad \textit{T. cordata} \qquad \text{(S4)}$$

g_s is then calculated based on a series of equations (detailed below) comprising physiological measurements and meteorological data following Tuzet's equation:

$$g_s = gmin + \frac{m\,(A_{net}+R)}{C_{i-}\,\Gamma} f_{\psi,leaf} \qquad \text{(S5)}$$

where g_{min} is the minimal stomatal conductance, m is a proportion factor between photosynthesis and stomatal conductance, C_i is the internal CO_2 concentration, and Γ is the CO_2 compensation point. The correction factor ($f_{\psi,leaf}$) is calculated as:

$$f_{\psi,leaf} = \frac{1+ e^{s_f \psi_f}}{1+ e^{s_f(\psi_f - \psi_{leaf})}} \quad \text{(S6)}$$

where S_f is the stomatal sensitivity and ψ_f is the reference water potential. Ψ_{leaf} was obtained with the following equation:

$$\psi_{leaf} = \psi_{soil}\ (R_{soil}/(R_{soil} + R_{root}))/(1\ /\ (R_{soil} + R_{root})) - TR * [(R_{soil} + R_{xylem} + R_{root})\ /\ f_{leaf,sun}] \quad \text{(S7)}$$

where ψ_{soil} is the soil water potential; R_{soil}, R_{root}, and R_{xylem} are soil, root, and xylem hydraulic resistances, and $f_{leaf\ sun}$ is the fraction of the leaves illuminated by the sun.

Next, the model calculates transpiration (TR) as:

$$TR = gs,max\ \frac{VPD}{P}\ LRR \quad \text{(S8)}$$

where LRR is the leaf root ratio and $g_{s,\ max}$ is the maximum stomatal conductance, computed from Farquar's equation depending on the limiting factor (minimum) between the electron transport rate (J) or carboxylation rate (V_C):

$$g_{s,max} = \frac{B\ (C_i - \Gamma) - R(DC_i + E)}{(E+F)(C_i - C_a)} \quad \text{(S9)}$$

where B is the CO_2 uptake limiting rate of either J_{max} or V_{Cmax}, Γ is the CO_2 compensation point of photosynthesis, E is a metric of carboxylation and oxygenation rates, and D and F are constants.

To consider the effect of T_{air} on J_{max} and V_{Cmax}, we adjusted the entropy factor (ΔS, J mol^{-1} K^1) and the activation energy (Ha, kJ mol^{-1}) in the peaked Arrhenius function with the general coefficients proposed by Kumarathunge *et al.* (2019) (Supplementary Table S8).

Once g_s from equation S5 is calculated, C_i is calculated from equation S1 and compared to the initial C_i. If the difference is important enough, a new iteration is conducted with updated g_s and C_i. Once C_i and g_s are stable, T_L is recalculated with the new gs and compared to the

previous T_L. Again, if the computed and initial T_L differ, the whole iteration process starts again with the new T_L until convergence.

Supplementary Section S2: $J_{max,25}$ and $V_{C,max,25}$ measurements

We measured $J_{max,25}$ and $V_{c,max,25}$ on five individuals of the three species once in July 2023 at our reference site of Cadenazzo (46°09'37.9"N; 8°56'00.7"E) with the Li-6800. To do so, we computed A/Ci curves by stepping down CO_2 inside the Li-6800 cuvette from 400 ppm to 300, 200, 100, 50, and 0 ppm and then returned to 400 ppm, then 400, 600, 800, 1000, 1200, 1500 and 2000 ppm CO_2. We fixed the air temperature in the cuvette to 25 °C, and the VPD to 1 and used a saturating light (PAR = 1500) during the measurements. We extracted $J_{max,25}$ and $V_{c,max,25}$ in R using the default method in the “plantecophys” package. All curves reached a saturating plateau and had at least 7 measurement points after removal of some outliers.

Supplementary Section S3: Minimum stomatal conductivity measurements

Leaf minimum conductance (g_{min}) was determined once in May 2022 using the mass loss of detached leaves method (Sack & Scoffoni, 2007) on 4-5 well-hydrated saplings per species growing at EPFL (46°31'15.3"N, 6°34'04.0"E, Lausanne, CH). Saplings were at the same physiological stage as those used for leaf gas exchange at the experimental sites. One small branch (or leaflet for *T. fortunei*) of each individual was sampled before dawn. One leaf per branch was sampled, sealed with melted candle wax on the exposed petiole, scanned with a flatbed scanner, weighed, and placed into a ziplock bag in a dark room with stable air temperature and humidity where temperature and humidity were tracked continuously with a sensor (HOBO MX2301A datalogger, Onset Computer Corporation, USA) (19.9°C & 66%, respectively). The fresh leaf mass was measured every 15 min with a precision balance until there were at least 8 points to observe the linear portion of the mass loss curve. G_{min} was then calculated as the transpiration rate divided by the VPD.

Supplementary Section S4: Tree growth, soil humidity, and chlorophyll content

At each campaign, we measured the height of each individual from the basis of the plant to the longest stem. We further measured the leaf chlorophyll content (CC) with a chlorophyll content meter (MC-100; Apogee Instruments; USA). As species-specific leaf anatomical structure can bias the measurements of the MC-100 (Parry, Blonquist and Bugbee, 2014), we calculated correction factors based on the protocol of Weiss (2014). For this, we measured 30 leaves of each species with the MC-100, then collected and stored the same leaves at -80 ºC until lyophilization as in Juillard *et al*. (2024) (Beta 2–8 LD plus; Martin Christ; Germany). We obtain the chlorophyll A and B concentration in µg ml^{-1} through repetitive ethanol dilution and measuring the absorbance of the solution with a spectrophotometer (Synergy Mx; Biotek; USA). We calculated CC per unit leaf area (µmol m^{-2}) by multiplying the raw CC by the number of dilutions and dividing by the specific leaf area (SLA) we had measured before lyophilization. All of these measurements were conducted to make sure the plants were in good condition and that sites and species were comparable.
